# The Associations Between Vegetarian and Vegan Diets and Orthorexia Nervosa Symptoms in Adults: A Systematic Review and Meta‐Analysis

**DOI:** 10.1002/eat.24596

**Published:** 2025-11-13

**Authors:** Valentina Díaz‐Goñi, Bruno Bizzozero‐Peroni, María Eugenia Visier‐Alfonso, Estela Jiménez‐López, Rubén Fernández‐Rodríguez, José Francisco López‐Gil, Tomás Olivo Martins‐de‐Passos, Alberto Durán González, Vicente Martínez‐Vizcaíno, Arthur Eumann Mesas

**Affiliations:** ^1^ Health and Social Research Center Universidad de Castilla‐La Mancha Cuenca Spain; ^2^ Aging Research Center, Department of Neurobiology, Care Sciences and Society Karolinska Institutet and Stockholm University Stockholm Sweden; ^3^ Higher Institute of Physical Education Universidad de la República Rivera Uruguay; ^4^ Center for Biomedical Research Network in Mental Health (CIBERSAM) Instituto de Salud Carlos III Madrid Spain; ^5^ Department of Physical Education and Sports, Faculty of Sports Science, Sport and Health University Research Institute (iMUDS) University of Granada Granada Spain; ^6^ Food & Mood Centre, Institute for Mental and Physical Health and Clinical Translation (IMPACT) Deakin University Geelong Australia; ^7^ The Grounded Minds Consortium A Deakin University & Flinder University Collaboration Victoria Australia; ^8^ School of Medicine Universidad Espíritu Santo Samborondón Ecuador; ^9^ Vicerrectoría de Investigación y Postgrado Universidad de Los Lagos Osorno Chile; ^10^ Postgraduate Program in Public Health Universidade Estadual de Londrina Londrina Brazil; ^11^ Facultad de Ciencias de la Salud Universidad Autónoma de Chile Talca Chile

**Keywords:** cross‐sectional, dietary patterns, disordered eating behavior, feeding and eating disorders, mental health, obsessive healthy eating, orthorexia, plant‐based diet, vegetarian

## Abstract

**Objective:**

To synthesize the evidence on the associations between vegetarian and/or vegan diets (VVDs) and symptoms of orthorexia nervosa (ON) compared with omnivorous diets in the adult population.

**Method:**

Following the Preferred Reporting Items for Systematic Reviews and Meta‐Analyses (PRISMA) and the Meta‐analyses of Observational Studies in Epidemiology (MOOSE) guidelines, we conducted a systematic review and meta‐analysis. We searched the MEDLINE/PubMed, Embase/Scopus, PsycINFO, and Web of Science databases up to June 17, 2025, with no language or date restrictions. Random effects models with the Sidik–Jonkman method were used to estimate pooled effect sizes.

**Results:**

The meta‐analysis included 26 cross‐sectional studies with a total of 23,783 participants (72.0% female; mean age range: 19.6–51.0 years). Adults who followed VVDs had moderately higher ON symptoms compared to omnivores (standardized mean differences using Cohen's d index = 0.46; 95% confidence interval [CI]: 0.33, 0.60; inconsistency index [*I* ^2^] = 81.0%). Additionally, categorical data revealed that VVD adherents were approximately twice as likely to report ON symptoms as omnivores (odds ratio = 1.99; 95% CI: 1.21–3.25; *I* ^2^ = 92.8%). Vegetarians and vegans were similarly associated with ON symptoms compared with omnivorous (*p* = 0.855).

**Discussion:**

Adherence to VVD is associated with higher ON symptoms in young and middle‐aged adults. However, these results should be interpreted with caution due to high heterogeneity and the low overall methodological quality of the exclusively cross‐sectional studies included. Higher‐quality longitudinal studies using validated assessment tools are needed to establish clearer causal relationships and inform clinical screening and intervention strategies.

**Summary**

- This is the first meta‐analysis to estimate the magnitude of the association between following vegetarian and/or vegan diets, compared to omnivorous diets, and symptoms of orthorexia nervosa.
- Higher adherence to vegetarian and/or vegan diets was associated with higher symptoms of orthorexia nervosa.
- These findings should be interpreted with caution due to the cross‐sectional design and overall low methodological quality of the available evidence, substantial between‐study heterogeneity, and limitations of the orthorexia assessment scales.

## 1 Introduction

The term “orthorexia” refers to an obsessive preoccupation with consuming healthy, “pure” and health‐promoting foods (Bratman 2017). Orthorexia nervosa (ON) is characterized by intense anxiety and distress due to extreme dietary restrictions, which can lead to changes in daily functioning (Horovitz and Argyrides 2023; Atchison and Zickgraf 2022). Although ON shares symptoms with some mental disorders including anorexia nervosa, bulimia nervosa, food avoidance/restriction disorders and obsessive‐compulsive disorders, such as restrictive eating, intrusive thoughts, and extreme meal planning, it has not been officially recognized as a disorder in the Diagnostic and Statistical Manual of Mental Disorders (DSM‐V‐TR) (Horovitz and Argyrides 2023; Niedzielski and Kaźmierczak‐Wojtaś 2021; Diagnostic and Statistical Manual of Mental Disorders 2022). A recent meta‐analysis of 30,476 individuals (61.2% female and aged 13–93 years) across 18 countries reported an ON symptoms prevalence of 27.5% (López‐Gil et al. 2023). However, this estimate may be inflated due to limitations in the screening tool (i.e., ORTO‐15) used to assess ON symptoms (Barrada and Meule 2024), as well as the lack of standardized diagnostic criteria (Niedzielski and Kaźmierczak‐Wojtaś 2021), which may lead to confusion between the pursuit of a healthier diet and ON (Gkiouleka et al. 2022).

The growing interest in healthy eating has been associated with the adoption of dietary restrictions, often leading individuals to follow dietary regimens that are perceived as more beneficial to health (Gortat et al. 2021), such as vegetarian and/or vegan diets (VVDs). These dietary patterns have grown in popularity because of their potential health and sustainability benefits (Hopwood et al. 2021). Vegetarian diets vary in strictness (Forestell et al. 2012), ranging from those that exclude meat but include eggs and/or dairy (i.e., ovo‐vegetarian, lacto‐vegetarian, and ovo‐lacto‐vegetarian diets) to those that avoid all animal products (i.e., vegan diets) (Hargreaves et al. 2023). The prevalence of VVDs varies substantially across regions. It generally ranges from 1% to 9% but can be as high as 36% in South Asia (Ruby 2012; Cramer et al. 2017).

VVDs have been associated with a lower risk of developing chronic conditions, such as cardiovascular disease, diabetes, hypertension, cancer, and dementia (Wang et al. 2023; Oussalah et al. 2020; Agnoli et al. 2023; Raj et al. 2025). However, these associations largely derive from observational studies and may be influenced by residual confounding, such as the clustering of healthier lifestyle behaviors (e.g., lower tobacco and alcohol use) among vegetarians (Gacek 2010). It is important to note that VVDs are not always associated with better health outcomes. In particular, strict vegan diets have been linked to an increased risk of hemorrhagic stroke, bone fractures and deficiencies in multiple vitamins and minerals (Wang et al. 2023). In addition, emerging evidence suggests that VVDs may be associated with adverse mental health outcomes in young and middle‐aged adults, including symptoms of depression and anxiety (Iguacel et al. 2021) and eating disorders (EDs) (McLean et al. 2022), while data on adolescents and older adults remain limited despite potential nutritional risks. The obsessive pursuit of healthy eating seen in individuals with ON, combined with the perceived health benefits of VVDs, may contribute to a reinforcing cycle in which restrictive dietary patterns both contribute to and are maintained by disordered eating behaviors (Szulc et al. 2025).

A previous narrative review on VVDs and ON suggested that individuals following these diets exhibit higher levels of ON symptoms than omnivores (Brytek‐Matera 2021). These results were recently confirmed by another narrative review (Szulc et al. 2025), suggesting a potential association between VVDs and ON, with evidence indicating that adopting such diets may sometimes serve to mask orthorexic behaviors. This overlap may be explained by shared characteristics, including selective food choices, rigid rules about “allowed” and “forbidden” foods, the central role of food in daily life, and inflexible eating patterns (Szulc et al. 2025). However, findings from two previous systematic reviews examining the relationship between VVDs and other EDs have been inconsistent. While some studies have identified an association between VVDs and EDs among adolescents and young adults (Sergentanis et al. 2020), others have reported mixed and inconclusive findings for both EDs and ON, suggesting that the way these diets are followed—rather than the diet itself—may constitute a risk factor for the development of disordered eating patterns (Mathieu et al. 2023). Despite these findings, a comprehensive and systematic synthesis of the literature on VVDs and ON symptoms is still lacking. Therefore, this systematic review and meta‐analysis synthesized evidence on the associations between VVDs and ON symptoms compared with omnivorous diets in the adult population.

## 2 Method

This systematic review with meta‐analysis was conducted according to the 2020 Preferred Reporting Items for Systematic Reviews and Meta‐Analyses (PRISMA) (Page et al. 2021) and the Meta‐analyses of Observational Studies in Epidemiology (MOOSE) (Sergentanis et al. 2020) guidelines (Stroup et al. 2000). The systematic review protocol was registered in the PROSPERO database (reference number: CRD42024527351). Two researchers (VDG and BBP) independently conducted the literature search, screening, study selection, data extraction, and methodological quality assessment. Any discrepancies were resolved through consultation with a third researcher (A.E.M.).

### 2.1 Search Strategy

The search process followed PRISMA‐S guidelines (Rethlefsen et al. 2021). A systematic search was conducted in the following electronic databases from the date of inception to September 9, 2024: MEDLINE (via PubMed), Embase (via Scopus), PsycINFO and the Web of Science Core Collection (via Clarivate's Web of Science). The search was subsequently updated to include the period from September 10, 2024 to June 17, 2025. Additional search methods were performed on Google Scholar along with citation searches (i.e., reviewing the references of the included studies and relevant systematic reviews [McLean et al. 2022; Mathieu et al. 2023; Brytek‐Matera 2021]). The search strategy included terms related to the objective of the study, including “vegetarian,” “vegan,” “plant‐based diets,” and “orthorexia.” The full approach to the search strategies is detailed in Table S1.

### 2.2 Eligibility Criteria

Eligibility criteria were based on the population, exposure, comparator, outcome (PECO) and study design (Morgan et al. 2018). To be included, studies had to report the following: (i) *population*: general adult population, with a reported mean age ≥ 18 years; (ii) *exposure*: vegetarian (i.e., ovo‐vegetarian, lacto‐vegetarian, or lacto‐ovo‐vegetarian) and/or vegan (i.e., exclusively plant‐derived foods) diets; (iii) *comparison*: omnivorous (nonvegetarian) diets that encompassed the consumption of any type of meat; (iv) *outcome*: ON symptoms, indicated by the number of symptoms (continuous outcome) or the presence of mild to severe symptoms (categorical outcome) using observer rating or self‐rating scales; and (v) *study design*: observational studies (i.e., cross‐sectional and longitudinal) published in peer‐reviewed academic journals. No language, publication date or other restrictions were applied.

Studies were excluded if they reported (i) data exclusively on populations with participants presenting specific pathologies (e.g., EDs, celiac disease) or conditions (e.g., athletes, pregnant); (ii) exposure diets categorized as vegetarian but consuming some type of meat (i.e., pescatarians, flexitarians, or semivegetarians); (iii) comparison diets classified as special diets (e.g., gluten free, paleo); (iv) ON data in conjunction with disordered eating symptoms or dietary restraint as outcomes; and (v) studies that reported results by individual items without an overall score.

### 2.3 Study Selection

All identified studies were uploaded to the Rayyan review system online (Ouzzani et al. 2016) and underwent deduplication. Two reviewers (V.D.G. and B.B.P.) subsequently conducted an independent title–abstract review, and studies deemed potentially eligible were then subjected to a full‐text screening to ascertain their compliance with the established eligibility criteria.

### 2.4 Data Extraction

Two independent reviewers (V.D.G. and B.B.P.) extracted the following information from each study: (i) first author surname, publication year and country; (ii) sample characteristics, including total sample size, mean age, sex assigned at birth and/or gender, race and/or ethnicity, socioeconomic status, body mass index (BMI), educational level (percentage of university degree), and subsamples according to diet type; (iii) exposure diets, including vegetarian, vegan, or mixed (vegetarian plus vegan) diets; (iv) measure of ON symptoms: ON symptoms by validated observer rating or self‐rating scales; and (v) main results, including ON symptoms as both continuous (means) and categorical (prevalence) data for each type of diet.

### 2.5 Methodological Quality Assessment

The National Institutes of Health (NIH) Quality Assessment Tool for Observational Cohorts and Cross‐Sectional Studies was used to assess the methodological quality of the included studies (National Heart Lung and Blood Institute 2021). This tool assesses 14 items across a range of domains, including the study population, the timeframe for associations, the exposure and outcome measures, and the statistical analyses. The responses to these domains (i.e., yes, no, not reported, not applicable, and cannot be determined) were used to inform the overall judgment. The quality of each study was evaluated according to the NIH quality rating guide (National Heart Lung and Blood Institute 2021), and each cross‐sectional study was rated as good (if most criteria were met), fair (if some criteria were met), or poor (if few criteria were met).

### 2.6 Effect Sizes

In this meta‐analysis, standardized mean differences (SMDs) determined via Cohen's *d* index (Cohen 1988) and odds ratios (ORs) with 95% confidence intervals (CIs) were used as the main effect sizes.

For continuous ON symptoms data, the means, standard deviations (SDs), and number of participants within each type of diet were extracted to estimate the SMDs. If the outcome data were reported as the means and CIs or standard errors, the SD was calculated with the appropriate formula following the Cochrane recommendations (Higgins et al. 2024). One study (Novara et al. 2022) reported outcome data as a beta coefficient, and the appropriate formula was used to determine the SMD (Borenstein et al. 2009). To estimate the pooled OR for categorical ON data, the number of participants and the cases within each type of diet were extracted. All effect sizes and main results of the included studies are listed in Table S2.

Positive SMD values indicate that individuals adhering to VVDs reported greater symptoms of ON than those following an omnivorous diet did. The criterion devised by Cohen was used to categorize the effect size estimator as either small (SMD = 0.2), moderate (SMD = 0.5), or large (SMD = 0.8) (Cohen 1988). OR values less than 1 indicate that individuals who followed a VVD had lower odds of ON than those who followed an omnivorous diet did.

### 2.7 Data Synthesis

The meta‐analyses were performed when at least five studies analyzed the outcome (Jackson and Turner 2017). A meta‐analysis of cross‐sectional studies comparing ON symptoms of VVDs with those of omnivorous diets was conducted via a random effects model with the Sidik–Jonkman method (Sidik and Jonkman 2005). Heterogeneity across studies was assessed via the inconsistency index [*I* ^2^], which was classified as not important (0%–40%), moderate (30%–60%), substantial (50%–90%), or considerable (75%–100%). The corresponding *p* values were also considered (Higgins et al. 2024).

Subgroup analyses were performed according to VVDs, geographic location, and self‐reported scales applied. Univariate mixed effects meta‐regression models were used to assess whether the characteristics of participants (i.e., age [years], sex [% females], BMI [kg/m^2^], and educational level [% university degree]) as continuous independent variables influenced the pooled estimate and between‐study heterogeneity. Furthermore, sensitivity analyses were performed to assess the robustness of the summary estimates via the leave‐one‐out method (Higgins et al. 2024). Publication bias was assessed by performing an Egger regression asymmetry test and visually inspecting funnel plots (Sterne et al. 2001).

Statistical significance was set at *p* < 0.05. All analyses were conducted via the Comprehensive Meta‐Analysis program (version 2; Biostat Inc.) and R software (version 4.2.3; R Foundation for Statistical Computing) with the *meta* (Balduzzi et al. 2019) package.

### 2.8 Additional Methodological Considerations

If the same study reported data from different ON scales (Ruiz and Quiles 2021) or from different motivations to follow a vegetarian diet (Dai and Leung 2024), the SMDs were pooled into a single result. When studies provided separate data for vegetarian and vegan diets (Barnett et al. 2016; Barthels et al. 2018; Coimbra and Ferreira 2021; Dunn et al. 2017; Ferreira and Coimbra 2021; Heiss et al. 2019; Missbach et al. 2015; Oberle et al. 2021; Yücel et al. 2021; Şentürk et al. 2022), these data were included in their respective subgroup analyses. In these cases, data on symptoms of ON were also combined to calculate a single pooled SMD for the inclusion of each study in the main meta‐analysis (VVDs vs. omnivorous diets). If ON scales reported scores inversely (i.e., higher score, higher ON symptoms vs. higher score, lower ON symptoms), we placed all effect sizes in a common frame (i.e., higher score, higher ON symptoms). When studies employed distinct cutoff points for establishing the prevalence of ON (Heiss et al. 2019), the data derived from the most severe cutoff point were included.

### 2.9 Studies With Missing Data

The first or corresponding authors of five studies (Ruiz and Quiles 2021; Missbach et al. 2015; Luck‐Sikorski et al. 2019; Parra‐Fernández et al. 2020; Brytek‐Matera 2020) were contacted at least twice via e‐mail to request substantial missing data for the study association (i.e., study sample according to diet type and prevalence of ON). Responses from three studies (Ruiz and Quiles 2021; Missbach et al. 2015; Brytek‐Matera 2020) provided sufficient data for inclusion, whereas the remaining two (Luck‐Sikorski et al. 2019; Parra‐Fernández et al. 2020) were classified as having missing data due to nonresponse.

## 3 Results

### 3.1 Study Selection

**FIGURE 1:**
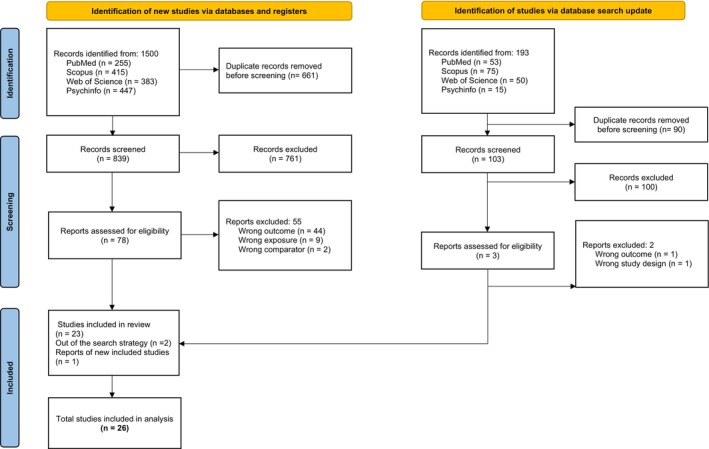
Flow diagram of study selection.

After removing duplicates, a total of 942 studies were eligible for title–abstract review, 81 of which were fully assessed for eligibility, and 57 were ultimately excluded for various reasons (Table S3). Two additional studies (Strahler et al. 2018; Tarsitano et al. 2022) were identified through complementary searches in Google Scholar and subsequently included. A total of 26 cross‐sectional studies were ultimately included in the systematic review and meta‐analysis (Figure 1).

### 3.2 Study Characteristics

Table 1 shows the main characteristics of the included studies. The 26 cross‐sectional studies were published between 2015 (Missbach et al. 2015) and 2025 (Albery et al. 2025). Of these, 15 (Novara et al. 2022; Dai and Leung 2024; Barnett et al. 2016; Barthels et al. 2018; Coimbra and Ferreira 2021; Dunn et al. 2017; Ferreira and Coimbra 2021; Missbach et al. 2015; Oberle et al. 2021; Yücel et al. 2021; Şentürk et al. 2022; Albery et al. 2025; Çiçekoğlu and Tunçay 2018; Dell'Osso et al. 2016; Hessler‐Kaufmann et al. 2020) reported continuous outcomes only, six (Luck‐Sikorski et al. 2019; Strahler et al. 2018; Tarsitano et al. 2022; Chard et al. 2019; Dittfeld et al. 2017; Gwioździk et al. 2022) reported categorical outcomes only, and five (Ruiz and Quiles 2021; Heiss et al. 2019; Reynolds et al. 2023; Dell'Osso et al. 2016; Białek‐Dratwa et al. 2024) reported both outcome types. Five studies (Çiçekoğlu and Tunçay 2018; Dittfeld et al. 2017; Gwioździk et al. 2022; Białek‐Dratwa et al. 2024; Brytek‐Matera et al. 2019) reported on the duration of adherence to VVD, but only one examined its relationship with ON (Dittfeld et al. 2017). Furthermore, only one study (Dai and Leung 2024) reported outcomes related to the ON diet according to the reason for choosing the diet. Two studies (Dittfeld et al. 2017; Reynolds et al. 2023) reported ON results according to different categories of vegetarian diets (ovo‐vegetarian, lacto‐vegetarian, and ovo‐lacto‐vegetarian). A paucity of studies (Çiçekoğlu and Tunçay 2018; Dell'Osso et al. 2016) have reported results according to sex concerning both VVDs and omnivorous diets. With respect to the instruments used to assess ON symptoms, 10 studies employed the Duesseldorf Orthorexia Scale (DOS) (Dai and Leung 2024; Barthels et al. 2018; Coimbra and Ferreira 2021; Ferreira and Coimbra 2021; Oberle et al. 2021; Luck‐Sikorski et al. 2019; Strahler et al. 2018; Tarsitano et al. 2022; Hessler‐Kaufmann et al. 2020; Chard et al. 2019), twelve studies utilized one of the versions of the Questionnaire for the Diagnosis of Orthorexia (ORTO‐15,ORTO‐11,ORTO‐R) (Ruiz and Quiles 2021; Barnett et al. 2016; Dunn et al. 2017; Heiss et al. 2019; Missbach et al. 2015; Yücel et al. 2021; Çiçekoğlu and Tunçay 2018; Dell'Osso et al. 2016, 2022; Hessler‐Kaufmann et al. 2020; Reynolds et al. 2023; Białek‐Dratwa et al. 2024), three studies employed the Teruel Orthorexia Scale (TOS) (Ruiz and Quiles 2021; Şentürk et al. 2022; Albery et al. 2025), one study used the Bratman Test for Orthorexia (BOT) (Dittfeld et al. 2017), and one study used the Eating Habits Questionnaire (EHQ) (Novara et al. 2022).

**TABLE 1:** Characteristics of the included studies.

Author, year, country	*N* (Total)	Age (mean years)	Sex assigned at birth and/or gender	Race/ethnicity (*n*)	SES	BMI	Education level (% university degree)	*n* (VVD/OMN)	Dietary exposition	Measure of orthorexia nervosa symptoms
Albery et al. (2025), UK	144	35	Female = 45.8%; male = 51.4; nonbinary = 2.8%	NR	NR	NR	NR	95/49	Vegetarian, vegan	TOS
Barnett et al. (2016), USA	284	38	Female = 83.4%	NR	NR	24.9	95	33/251	Vegetarian, vegan	ORTO‐15
Barthels et al. (2018), Germany	351	32	Female = 63.2%	NR	NR	23.7	NR	177/174	Vegetarian, vegan	DOS
Białek‐Dratwa et al. (2024), Poland	186	> 18	Female = 94.0%; male = 5.9%	NR	NR	NR	51	84/102	Vegetarian, vegan	ORTO‐15
Chard et al. (2019), USA	384	19.6	Female = 69.5%; male = 30.5%	NR	NR	23	100	30/256	Vegetarian	DOS
Çiçekoğlu and Tunçay (2018), Turkey	62	34	Female = 61.0%	NR	NR	NR	NR	31/31	Vegetarian, vegan	ORTO‐11
Coimbra and Ferreira (2021), Portugal	447	32	Female = 100.0%	NR	NR	24	R	111/281	Vegetarian, vegan	DOS
Dai and Leung (2024), USA	334	> 18	Female = 58.9%; male = 38.9%; nonbinary = 0.6%; prefer not to say = 1.5%	White = 264 Black = 30 Hispanic = 18 American Indian or Alaska Native = 1 Asian = 15 Native Hawaiian or Pacific Islander = 0 Other = 6	≤ $25,000 = 15.6% $25,000–$49,999 = 26.3% $50,000–$74,999 = 25.1% $75,000–$99,999 = 12.3% $100,000–$149,999 = 9.9% ≥ $150,000 = 8.4% Prefer not to say = 2.4%	NR	NR	230/104	Vegetarian	DOS
Dell'Osso et al. (2016), Italy	2826	29	Female = 40.6%; male = 59.4%	NR	NR	22.5	75	313/2513	Vegetarian	ORTO‐15
Dell'Osso et al. (2022), Italy	2140	24	Female = 66.0%; male = 33.9%	NR	NR	NR	100	118/1956	Vegetarian, vegan	ORTO‐R
Dittfeld et al. (2017), Italy	2611	24	NR	NR	NR	22.1	47	1346/1265	Vegetarian	BOT
Dunn et al. (2017), USA	275	22	Female = 68.0%; male = 31.0%	White = 214 Latino = 41 African American = 11 Asian American = 5 Declined to answer = 4	NR	NR	100	34/219	Vegetarian	ORTO‐15
Ferreira and Coimbra (2021), Portugal	541	35	Female = 82.6%	NR	NR	24.4	NR	126/357	Vegetarian, vegan	DOS
Gwioździk et al. (2022), Poland	420	24	Female = 100.0%	NR	NR	NR	88	115/227	Vegetarian	ORTO‐15
Hessler‐Kaufmann et al. (2020), Germany	511	43	Female = 63.4%	NR	NR	25	51	49/364	Vegetarian	DOS
Heiss et al. (2019), USA	381	31	Female = 80.8%	White = 335	NR	25	NR	241/106	Ovo‐lacto vegetarian, vegan	ORTO‐15
Luck‐Sikorski et al. (2019), Germany	1007	51	Female = 48.6%	NR	NR	26	NR	59/948	Vegetarian	DOS
Missbach et al. (2015), Austria	1029	31	Female = 74.6%; male = 25.4%	NR	NR	23	37	179/829	Vegetarian, vegan	ORTO‐15
Novara et al. (2022), Italy	1075	21	Female = 75.0%	NR	NR	NR	NR	58/1017	Vegetarian, vegans	EHQ
Oberle et al. (2021), USA	847	22	Female = 82.0%; male = 15.0%	NR	NR	NR	47	120/606	Vegetarian, vegan	DOS
Yücel et al. (2021), Turkey	965	20–65	Female = 76.0%; male = 23.7%	NR	Employed = 319 unemployed = 607; left work = 39	NR	NR	603/362	Vegetarian, vegan	ORTO‐15
Reynolds et al. (2023), USA, India, Australia, UK, Canada, Others.	444	37	Female = 45.5%; male = 53.4%; other = 0.7%	Caucasian = 254 Southeast Asian = 42; other = 60; East Asian = 33; African = 19; Latino/Hispanic = 11; South Asian = 10; mixed = 6; missing = 6; Middle Eastern = 3	NR	27	59	70/374	Vegetarian, vegan, ovo‐lacto vegetarian, ovo‐vegetarian, lacto‐vegetarian	ORTO‐15
Ruiz and Quiles (2021), Spain	534	22	Female = 79.0%; male = 21.0%	NR	NR	NR	100	86/448	Vegetarian, vegan	ORTO‐11, TOS
Şentürk et al. (2022), Turkey	1165	31	Female = 81.5%; male = 17.0%; other = 1.5%	NR	NR	22.3	NR	739/426	Vegetarian, vegan	TOS
Strahler et al. (2018), Germany	713	29	Female = 58.0%	NR	Employment: student/unemployed = 238; full time = 153; part time = 122; mini job = 134; nonregular job = 55; parental leave = 11	23	94	140/451	Vegetarian, vegan	DOS
Tarsitano et al. (2022), Italy	4107	31	Female = 95.0%	NR	NR	24	NR	1071/2931	Vegetarian, vegan	DOS

Abbreviations: BMI, body mass index; BOT, Bratman Test for Orthorexia; DOS, Duesseldorf Orthorexia Scale; EHQ, Eating Habits Questionnaire; NR, not reported; OMN, omnivorous diets; ORTO‐15/11, Questionnaire for the Diagnosis of Orthorexia; ORTO‐R, abbreviated version of the ORTO‐11; SES, socioeconomic status; TOS, Teruel Orthorexia Scale; VVD, vegetarian and/or vegan diet.

### 3.3 Participants

The studies included a total of 23,783 individuals (72.0% females) with a mean age ranging from 19.6 (Chard et al. 2019) to 51.0 (Luck‐Sikorski et al. 2019) years and a mean BMI ranging from 22.1 (Dittfeld et al. 2017) to 27.0 kg/m^2^ (Reynolds et al. 2023) at baseline. The participants were recruited from diverse geographical locations, including Australia (Missbach et al. 2015; Reynolds et al. 2023), Canada (Reynolds et al. 2023), Germany (Barthels et al. 2018; Luck‐Sikorski et al. 2019; Strahler et al. 2018; Hessler‐Kaufmann et al. 2020), India (Reynolds et al. 2023), Italy (Novara et al. 2022; Dell'Osso et al. 2016, 2022; Dittfeld et al. 2017; Tarsitano et al. 2022), Poland (Białek‐Dratwa et al. 2024), Portugal (Coimbra and Ferreira 2021; Ferreira and Coimbra 2021), Spain (Ruiz and Quiles 2021), Turkey (Yücel et al. 2021; Şentürk et al. 2022; Çiçekoğlu and Tunçay 2018), the United Kingdom (Albery et al. 2025; Reynolds et al. 2023), and the United States (Dai and Leung 2024; Barnett et al. 2016; Dunn et al. 2017; Heiss et al. 2019; Oberle et al. 2021; Chard et al. 2019; Reynolds et al. 2023). The studies included data from vegetarians (Luck‐Sikorski et al. 2019; Chard et al. 2019; Dittfeld et al. 2017; Gwioździk et al. 2022), vegetarians and vegans combined (Ruiz and Quiles 2021; Dai and Leung 2024; Tarsitano et al. 2022; Çiçekoğlu and Tunçay 2018; Dell'Osso et al. 2016, 2022; Hessler‐Kaufmann et al. 2020; Reynolds et al. 2023; Białek‐Dratwa et al. 2024), and vegetarians and vegans separately (Novara et al. 2022; Barnett et al. 2016; Barthels et al. 2018; Coimbra and Ferreira 2021; Dunn et al. 2017; Ferreira and Coimbra 2021; Heiss et al. 2019; Missbach et al. 2015; Oberle et al. 2021; Yücel et al. 2021; Şentürk et al. 2022; Strahler et al. 2018; Albery et al. 2025).

### 3.4 Methodological Quality of the Included Studies

According to the NIH Quality Assessment Tool (National Heart Lung and Blood Institute 2021), cross‐sectional studies were scored between 3 and 7 points (no studies were rated as good quality, 15.4% were rated as fair quality, and 84.6% were rated as poor quality). Most studies had methodological limitations on six key criteria: (i) justified sample size; (ii) exposure assessed prior to outcome; (iii) sufficient time frame; (iv) valid measure of exposure; (v) repeated exposure assessment; and (vi) blinding of outcome assessors to participants' exposure status. (Table S4).

### 3.5 Meta‐Analysis

**FIGURE 2:**
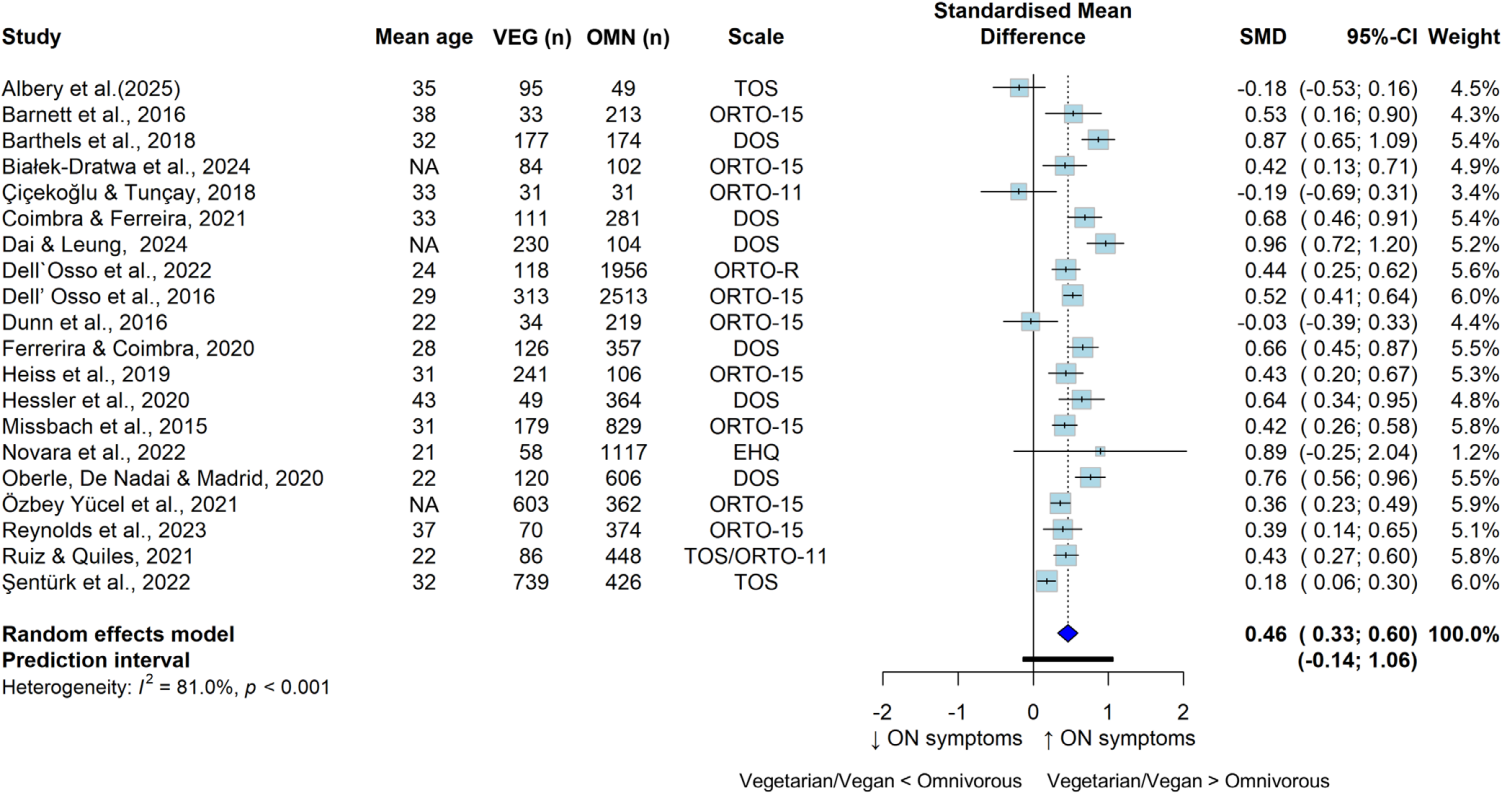
Pooled standardized mean differences for the cross‐sectional associations between vegetarian and/or vegan versus omnivorous diets and orthorexia nervosa symptoms. CI, confidence interval; DOS, Duesseldorf Orthorexia Scale; EHQ, Eating Habits Questionnaire; OMN, omnivorous; ON, orthorexia nervosa; ORTO‐15/11/R, Questionnaire for the Diagnosis of Orthorexia; SMD, standardized mean difference; TOS, Teruel Orthorexia Scale; VEG, vegetarian and/or vegan.

**FIGURE 3:**
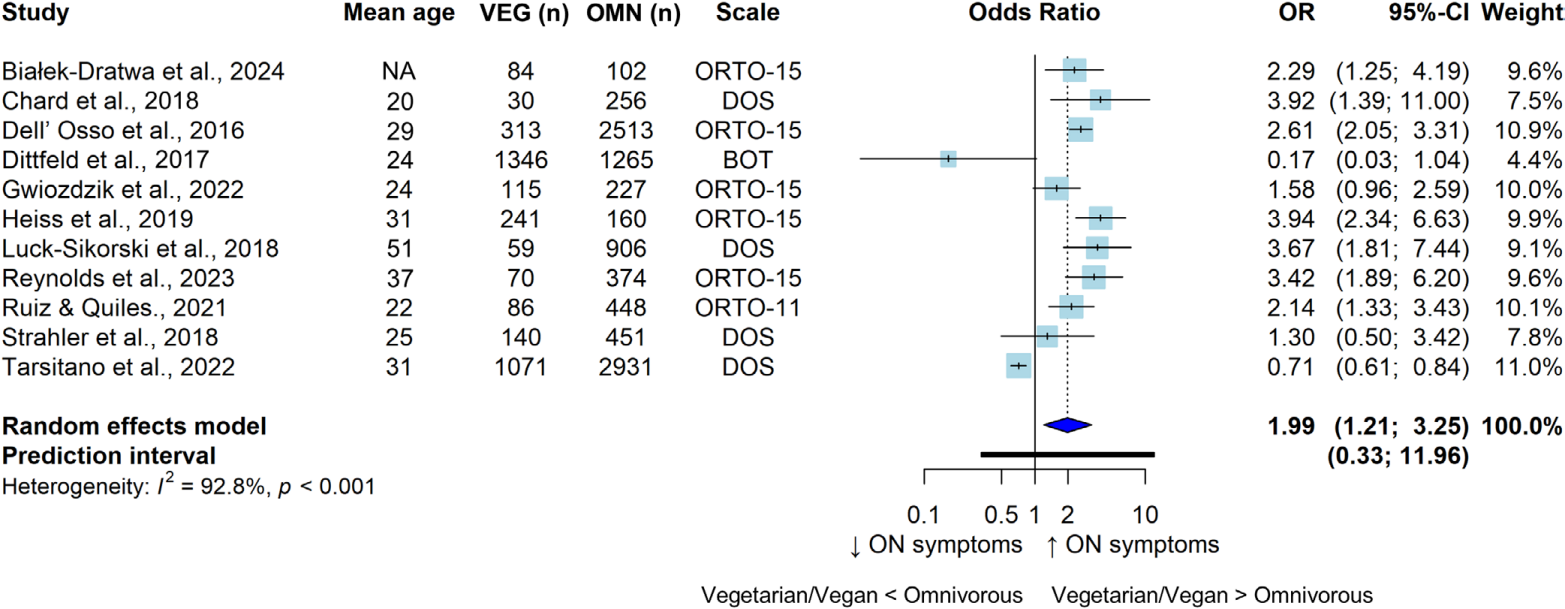
Pooled odds ratios for the cross‐sectional associations between vegetarian and/or vegan versus omnivorous diets and the likelihood of orthorexia nervosa symptoms. BOT, Bratman Test for Orthorexia; CI, confidence interval; DOS, Duesseldorf Orthorexia Scale; OMN, omnivorous; ON, orthorexia nervosa; OR, odds ratio; ORTO‐15/11, Questionnaire for the Diagnosis of Orthorexia; VEG, vegetarian and/or vegan.

The meta‐analyses according to continuous or categorical ON data are displayed in Figures 2 and 3, respectively. Compared with those following omnivorous diets (*n* = 10,631), adults following VVDs (*n* = 3497) presented higher ON symptoms (SMD = 0.46; 95% CI: 0.33, 0.60; *I* ^2^ = 81.0%; *n* = 20). The meta‐analysis of categorical data revealed a greater prevalence of ON symptoms (OR = 1.99; 95% CI: 1.21, 3.25; *I* ^2^ = 92.8%; *n* = 11) among adults following VVDs (*n* = 3555; cases: 677) than among those following omnivorous diets (*n* = 9633; cases: 2013).

#### 3.5.1 Subgroup Analyses and Meta‐Regression Models

**FIGURE 4:**
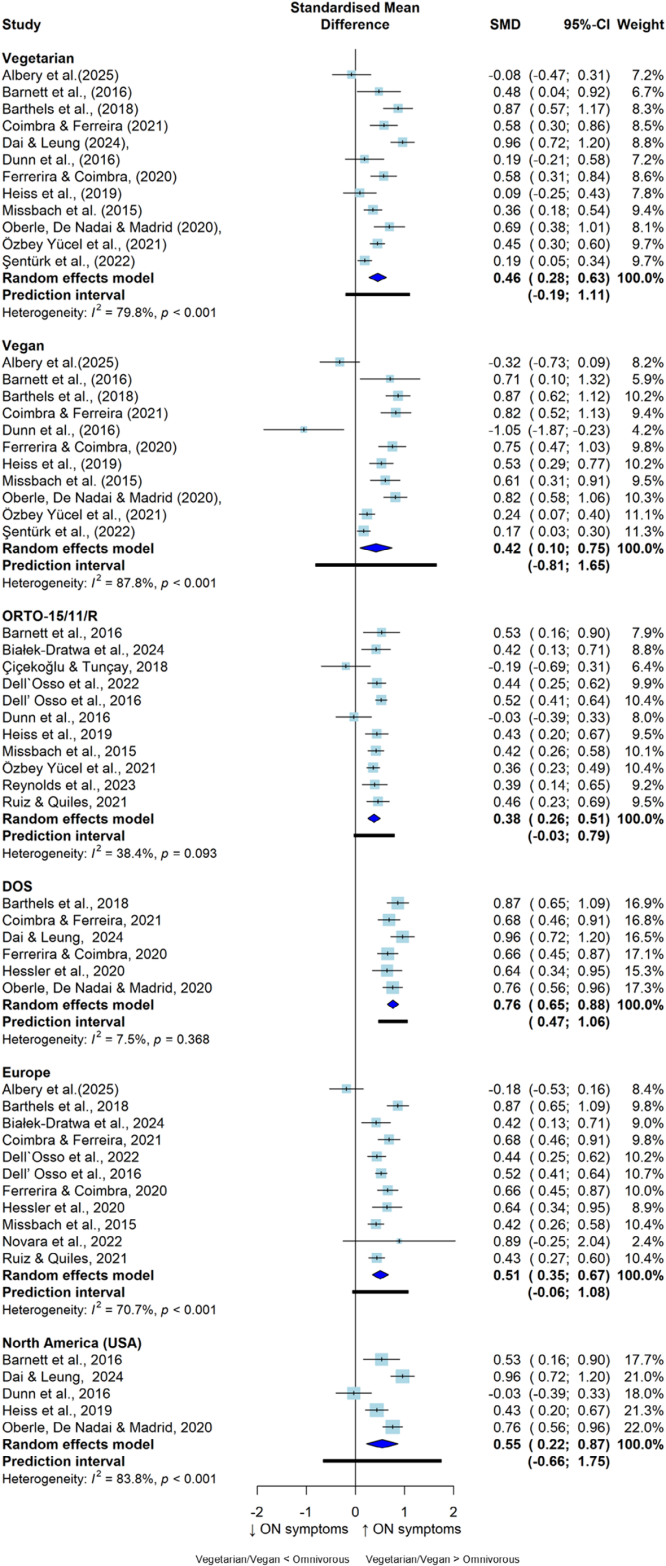
Pooled standardized mean differences between vegetarian and/or vegan versus omnivorous diets and orthorexia nervosa symptoms, by subgroup. CI, confidence interval; DOS, Duesseldorf Orthorexia Scale; ON, orthorexia nervosa; ORTO‐15/11/R, Questionnaire for the Diagnosis of Orthorexia; SMD, standardized mean difference.

The results of the subgroup analyses are displayed in Figure 4 and Table S5. Compared with omnivorous diets, vegetarian (SMD = 0.46; 95% CI: 0.28, 0.63; *I* ^2^ = 79.8%; *n* = 12) and vegan (SMD = 0.42; 95% CI: 0.10, 0.75; *I* ^2^ = 87.8%; *n* = 11) diets were moderately associated with higher ON symptoms. No significant subgroup differences were found between vegetarians and vegans in their associations with ON symptoms (*p* = 0.855).

Compared with those following omnivorous diets, ON symptoms were significantly greater for adults following VVDs according to the self‐reported scales applied. Specifically, studies employing the ORTO‐15/11/R questionnaire reported a small to moderate effect size (SMD = 0.38; 95% CI: 0.26, 0.51; *I* ^2^ = 38.4%; *n* = 11), and studies employing the DOS reported a large effect size (SMD = 0.76; 95% CI: 0.65, 0.88; *I* ^2^ = 7.5%; *n* = 6), with significant between‐group differences (*p* < 0.001). Additionally, the subgroup analysis according to geographic location revealed no significant differences (*p* = 0.838) between studies from the USA (SMD = 0.55; 95% CI: 0.22, 0.87; *I* ^2^ = 83.8%; *n* = 5) and Europe (SMD = 0.51; 95% CI: 0.35, 0.67; *I* ^2^ = 70.7%; *n* = 11). Owing to the small number of studies (*n* < 5) analyzing categorical outcome data in specific subgroups (i.e., USA, vegan, DOS), subgroup analyses were not performed for the meta‐analysis of the prevalence of ON symptoms between VVDs and omnivorous diets.

Meta‐regression analyses for participant characteristics revealed no statistically significant differences in the study associations (Table S6 and Figure S1).

#### 3.5.2 Sensitivity Analyses and Publication Bias

The results of the sensitivity analysis are shown in Table S7. The pooled SMDs were not modified when each study was removed one by one. According to Egger's test (Table S8) and funnel plot asymmetry, no publication bias was found for the study associations (*p* = 0.762 for continuous outcome data and *p* = 0.121 for categorical outcome data) (Figure S2).

## 4 Discussion

To the best of our knowledge, this systematic review and meta‐analysis is the first to synthesize observational evidence on the associations between VVDs and symptoms of ON in young and middle‐aged adults. Our findings suggest that VVDs followers are more likely to exhibit ON symptoms than omnivores. Subgroup analyses revealed that both VVDs were moderately associated with greater ON symptoms than were omnivorous diets, although there was no significant difference between the two. Notably, the magnitude of the association varied depending on the self‐report instrument used. Studies using the ORTO‐15/11/R questionnaire showed a small‐to‐moderate effect size, whereas those using the DOS reported a large effect size. No significant differences were observed by geographic location or across participant characteristics assessed in the meta‐regression models, including age, sex, BMI, and educational level. However, our findings should be interpreted with caution due to important limitations, including the lack of longitudinal studies–particularly prospective cohort studies– in the available literature and the generally low methodological quality of the included studies.

While previous reviews were limited to systematic syntheses (McLean et al. 2022; Mathieu et al. 2023), the present study not only confirms their main findings of a positive association between VVDs and ON symptomatology but also contributes to the field in two important ways. First, it provides the first meta‐analytic estimate of this association by directly comparing individuals following VVDs with those adhering to omnivorous diets. Second, it extends the evidence through subgroup analyses and meta‐regressions to explore potential moderating factors. These analyses reinforce the general finding that VVDs are moderately associated with an increased likelihood of ON symptoms compared with omnivorous diets. Interestingly, no significant differences were detected between vegetarians and vegans. These associations may be partially explained by the restrictive and selective eating patterns characteristic of certain VVDs, which align with potential behavioral markers of ON (Şentürk et al. 2022; Horovitz and Argyrides 2023). The significant heterogeneity observed, particularly regarding vegan diets, underscores the impact of methodological and contextual factors.

Some symptoms of ON, such as disordered restrictive eating, the pursuit of thinness, and weight control, are shared with other EDs (Atchison and Zickgraf 2022). These features are like the established relationships between VVDs and other EDs, particularly anorexia nervosa (Sergentanis et al. 2020). For individuals following a VVD and living with an ED, this dietary pattern may serve as a form of food restriction that either reflects or reinforces their underlying psychopathology (Lindeman et al. 2000). However, owing to the predominance of cross‐sectional data, the directionality of the relationship between VVDs and ON cannot be established.

The VVD‐ON association may be influenced by multiple factors, including psychological characteristics (e.g., personality traits, perfectionism, and obsessive‐compulsive tendencies), sociocultural influences (e.g., excessive social media engagement, exposure to misinformation, and body stereotypes), and a history of chronic dieting and body image dissatisfaction (Horovitz and Argyrides 2023), which should be considered in future research.

In addition to individual traits and behavioral patterns, dietary motivations may influence the relationship between VVDs and ON symptoms (Szulc et al. 2025). A deeper understanding of the motivations behind adopting VVDs could provide more precise insights into the temporality of this relationship. For example, some studies have reported a decrease in ON symptoms among individuals who follow these diets due to concerns about animal welfare, environmental issues, or political beliefs (Mathieu et al. 2023). Thus, future studies must explore the reasons behind these dietary choices to further examine their associations with EDs.

Another possible explanation for the association between VVDs and ON symptoms is that, despite efforts to develop criteria for identifying risk factors and assessing appropriate treatment (Dunn and Bratman 2016; Moroze et al. 2015), a wide variety of instruments have been used to assess ON symptoms. Most of these instruments have been criticized for their validity in detecting these features and screening for ON. For example, ORTO‐15, one of the most widely used tools, has been shown to be unreliable by some authors (López‐Gil et al. 2023; Ng et al. 2024; Depa et al. 2019). This suggests that the high prevalence of ON (ranging from 30% to 70%) may be due to the variety of available measurement instruments, as well as their low reliability (Dunn and Bratman 2016; Barrada and Meule 2024). Therefore, future research should prioritize the use of validated tools that are more accurate and reliable (e.g., DOS, TOS and EHQ‐R) (Opitz et al. 2020). This will enable more consistent and comparable estimates to be obtained across studies. Consequently, certain behaviors may be misclassified as pathological when they may reflect a nonproblematic, health‐oriented interest in eating. This underscores the necessity of further research into the etiology of this condition, with the aim of establishing standardized criteria and accurate assessment tools (Horovitz and Argyrides 2023; Donini et al. 2022).

### 4.1 Strengths, Limitations, and Future Recommendations

An important strength of our study is that searching multiple databases exhaustively increased the likelihood of capturing all available evidence on the subject, thereby improving the completeness and reliability of the results. However, some limitations must also be noted. First, the inclusion of only cross‐sectional studies prevented causal inferences and definitive conclusions about whether VVDs lead to the development of ON symptoms. Second, our analyses revealed substantial between‐study heterogeneity in the main pooled estimates, partially attributable to differences in geographic region, the instruments used to assess ON symptoms, and the influence of specific low‐quality studies (Şentürk et al. 2022; Tarsitano et al. 2022). Nevertheless, a considerable proportion of heterogeneity remained unexplained, which limits the interpretability of the findings. Third, the small number of studies with sex‐stratified data prevented us from examining potential differences in the VVD–ON association. Fourth, most studies did not report sociodemographic characteristics, such as ethnicity, race, gender, and socioeconomic status. This lack of information limits the ability to evaluate the representativeness and diversity of the samples, as well as the generalizability of the findings to broader populations. Future studies should prioritize systematically reporting these characteristics to enable stratified meta‐analyses and meta‐regression and improve the external validity of the evidence. Finally, most studies failed to account for potentially influential covariates, such as sex, physical activity, socioeconomic status, energy intake, the reason for adhering to VVDs, the duration of adherence to these diets, and the use of social networks. This limited our ability to perform adjusted analyses for these factors. Future studies should consider key factors that may influence this relationship, such as the underlying reasons for adopting these diets (e.g., religious, ethical or environmental reasons), the duration of adherence to these diets, specific subtypes of vegetarian diets (e.g., ovo‐vegetarian, lacto‐vegetarian, and ovo‐lacto‐vegetarian diets), the nutritional quality of the diet, and the individual characteristics of the participants. The results from these analyses should also be reported by sex and BMI. It is important to distinguish between pathological behaviors, such as those associated with ON, and nonpathological behaviors that promote healthy eating. This distinction would contribute to clarifying their respective associations with dietary patterns. Research on specific age groups, particularly adolescents and older adults, who may have different vulnerabilities and motivations in relation to eating behaviors, is lacking.

## 5 Conclusion

Young and middle‐aged adults who follow VVDs appear to exhibit more ON symptoms than omnivores. However, these findings should be interpreted with caution because the current evidence is limited to cross‐sectional studies, which preclude causal inference. Additionally, the studies are generally of low methodological quality. Future high‐quality research should prioritize longitudinal designs, the use of standardized and validated assessment tools, and improved methodological rigor to better understand the nature and direction of the association between VVDs and ON symptomatology. This information could help identify individuals who could benefit from early screening and tailored interventions, particularly in clinical and nutritional counseling contexts.

## Author Contributions

**Valentina Díaz‐Goñi:** conceptualization (lead), investigation (lead), methodology (equal), formal analysis (equal), visualization (lead), writing – original draft preparation (lead), writing – review and editing (equal). **Bruno Bizzozero‐Peroni:** conceptualization (supporting), investigation (supporting), methodology (equal), formal analysis (equal), writing – original draft preparation (supporting), visualization (supporting), validation (lead), project administration (equal), writing – review and editing (equal), supervision (equal). **María Eugenia Visier‐Alfonso:** validation (supporting), writing – review and editing (equal). **Estela Jiménez‐López:** validation (supporting), writing – review and editing (equal). **Rubén Fernández‐Rodríguez:** writing – original draft preparation (supporting), writing – review and editing (equal). **José Francisco López‐Gil:** writing – original draft preparation (supporting), writing – review and editing (equal). **Tomás Olivo Martins‐de‐Passos:** visualization (supporting), writing – review and editing (equal). **Alberto Durán González:** visualization (supporting), writing – review and editing (equal). **Vicente Martínez‐Vizcaíno:** methodology (supporting), writing – review and editing (equal). **Arthur Eumann Mesas:** conceptualization (supporting), investigation (supporting), methodology (equal), writing – original draft preparation (supporting), visualization (supporting), project administration (equal), writing – review and editing (equal), supervision (equal). All the authors have read and agreed to the published version of the manuscript.

## Ethics Statement

The authors have nothing to report.

## Consent

The authors have nothing to report.

## Conflicts of Interest

The authors declare no conflicts of interest.

## Supporting information


**Table S1:** Detailed information on search strategies.
**Table S2:** Effect sizes, main results, and covariate adjustments of included studies.
**Table S3:** List of the studies fully assessed for eligibility and excluded.
**Table S4:**. Methodological quality of included studies^a^.
**Table S5:** Subgroup analyses for the cross‐sectional associations between vegetarian and/or vegan versus omnivorous diets and orthorexia nervosa symptoms.
**Table S6:** Meta‐regression analyses for the cross‐sectional associations between vegetarian and/or vegan versus omnivorous diets and orthorexia nervosa symptoms. ^a^
**Table S7:**. Sensitivity analyses excluding studies one by one.
**Table S8:** Meta‐bias for the cross‐sectional associations between vegetarian and/or vegan versus omnivorous diets and orthorexia nervosa symptoms.
**Figure S1:** Meta‐regression models by age (A), sex (B), body mass index (C), and educational level (D) for the cross‐sectional associations between vegetarian and/or vegan versus omnivorous diets and orthorexia nervosa symptoms.
**Figure S2:** Publication bias from cross‐sectional meta‐analyses of standardized mean differences (A) and odds ratio (B).

## Data Availability

The data that support the findings of this study are available from the corresponding author upon reasonable request.
