# Supplementary material for: The Associations Between Vegetarian and Vegan Diets and Orthorexia Nervosa Symptoms in Adults: A Systematic Review and Meta‐Analysis
Source: Int J Eat Disord. 2025 Nov 13;59(3):409–23. doi: 10.1002/eat.24596 (PMC12979968; doi:10.1002/eat.24596)

**Supplementary Material**

**The Associations between Vegetarian and Vegan Diets and Orthorexia Nervosa Symptoms in Adults: A Systematic Review and Meta-Analysis**

**Supplementary Table 1.** Detailed information on search strategies.

| **Information source** | **Method** |
| --- | --- |
| Databases^a^: PubMed^b^, Scopus^c^,Web of Science^c^, PsycINFO^c^ | (vegetarian* OR vegan* OR plant-based OR "vegetable diet" OR Adventist OR meat-free OR “meat avoidance” OR “nonmeat eat*” OR “plant-rich diet”) AND (orthorex* OR “patholog* eating” OR “eating patholog*” OR “avoidant food” OR “feeding disorder*” OR “extreme diet*” OR “eating disorder*” OR “disorder* eating” OR “eating behavio*” OR “eating symptom*” OR “eating attitude*” OR “eating problem*” OR ORTO) |
| Online resources and browsing | Portal (URL): Google Scholar ([https://scholar.google.com](https://scholar.google.com/)). Search terms: “vegetarian diet” AND orthorexia. The first 100 records (n=10 pages) were selected and screened. |
| Citation searching | Relevant systematic reviews were kept separate to screen their included studies^1,2,3^.  References of included articles were manually screened to identify additional studies. |

^a^ Data were retained in Research Information Systems (RIS) or PubMed formats for each database. ^b^ Search strategy restricted to Title/Abstract. ^c^ Search strategy restricted to Title/Abstract/Keywords.

References cited in this table:

^1^ Mathieu S, Hanras E, Dorard G. Associations between vegetarianism, body mass index, and eating disorders/disordered eating behaviours: a systematic review of literature. Int J Food Sci Nutr 2023; 74: 424–462.

^2^ McLean CP, Kulkarni J, Sharp G. Disordered eating and the meat-avoidance spectrum: a systematic review and clinical implications. Eating and Weight Disorders 2022; 27: 2347–2375.

^3^ Brytek-Matera A. Vegetarian diet and orthorexia nervosa: a review of the literature. Eating and Weight Disorders 2021; 26: 1–11

**Supplementary Table 2.** Effect sizes, main results, and covariate adjustments of included studies.

| **Reference** | **Measure of ON symptoms** | **Effect size** | **Vegetarians** | **Vegans** | | **Omnivores** | | | **Covariate adjustment** | |
| --- | --- | --- | --- | --- | --- | --- | --- | --- | --- | --- |
| Albery et al., 2025 | TOS | Mean (SD) | 8.18±3.77 | | 7.38±3.68 | | 8.45±3.03 | - | |  |
| Barnett et al., 2016 | ORTO-15 | Mean (SD) | 35.02 ± 4.12 | | 34.18 ± 3.66 | | 36.85 ± 3.79 | - | |  |
| Barthels et al., 2018 | DOS | Mean (SD) | 20.56 ± 4.70 | | 20.96 ± 6.04 | | 16.09 ± 5.29 | - | |  |
| Bialek-Dratwa et al., 2024 | ORTO-15 | Mean (SD) | 35.76 ± 3.50 | | - | | 37.24 ± 3.50 | - | |  |
|  | ORTO-15 (≥35) | Prevalence, n (%) | 41 (48.8) | | - | | 30 (29.4) | - | |  |
| Chard et al., 2018 | DOS (≥30) | Prevalence, n (%) | 6 (20) | | - | | 15 (6) | - | |  |
| Çiçekoğlu & Tunçay, 2018 | ORTO-11 | Mean (SD) | 27.70 ± 2.80 | | - | | 27.10 ± 3.40 | - | |  |
| Coimbra & Ferrerira, 2021 | DOS | Mean (SD) | 20.07 ± 5.38 | | 21.76 ± 7.35 | | 16.69 ± 5.92 | - | |  |
| Dai & Leung, 2024 | DOS | Mean (SD) | 2.69 ± 0.69 | | - | | 2.05 ± 0.61 | Age, gender, ethnicity, household income, urbanicity, physical health | |  |
| Dell’Osso et al., 2016 | ORTO-15 | Mean (SD) | 35.23 ± 4.81 | | - | | 37.37 ± 3.98 | - | |  |
|  | ORTO-15 (≥35) | Prevalence, n (%) | 166 (53) | |  | | 759 (30.2) | - | |  |
|  | ORTO-15 | OR (95% CI) | 2.51 (1.97– 3.20) | | - | | Reference | Age, gender, education, professional role, parent educational  level, appreciation  of the project, weight | |  |
| Dell`Osso et al., 2022 | ORTO-R | Mean (SD) | 14.19 ± 3.40 | | - | | 12.63 ± 3.59 | Gender, type of diet, and presence  of autistic  traits | |  |
| Dittfeld et al., 2017 | BOT (≥10) | Prevalence, n (%) | 1 (0.1) | | - | | 8 (0.6) | - | |  |
| Dunn et al., 2016 | ORTO-15 | Mean (SD) | 36.50 ± 4.40 | | 42.50 ± 1.00 | | 37.40 ± 4.90 | - | |  |
| Ferrerira & Coimbra, 2021 | DOS | Mean (SD) | 20.39 ± 5.31 | | 21.65 ± 7.29 | | 16.94 ± 6.08 | - | |  |
| Gwiozdzik et al., 2022 | ORTO-15 (≥35) | Prevalence, n (%) | 38 (33) | | - | | 54 (23.8) | - | |  |
| Hessler et al., 2021 | DOS | Mean (SD) | 19.00 ± 5.00 | | - | | 16.00 ± 4.60 | Age, sex, BMI | |  |
| Heiss et al., 2019 | ORTO-15 | Mean (SD) | 36.96 ± 4.30 | | 35.2 ± 4.06 | | 37.33 ± 3.96 | BMI, gender | |  |
|  | ORTO-15 (≥40) | Prevalence, n (%) | 38 (76) | | 161 (84.3) | | 73 (68.9) |  |  |  |
| Luck‑Sikorski et al., 2019 | DOS (≥30) | Prevalence, n (%) | 19 (11) | | - | | 6 (54) | - | |  |
|  | DOS (≥30) | OR (95% CI) | 4.37 (1.47–12.99) | | - | | Reference | Age, gender, school, depressive symptoms, BMI, allergies | |  |
| Missbach et al., 2015 | ORTO-15 | Mean (SD) | 23.47 ± 3.64 | | 22.6 ± 3.82 | | 24.72 ± 3.47 | - | |  |
| Novara et al., 2022 | EHQ | β (95% CI) | 0.08 (0.04- 0.13) | | 0.15 (0.06-0.25) | | Reference | Eating disorder risk | |  |
| Oberle et al., 2021 | DOS | Mean (SD) | 47.21 ± 15.75 | | 48.78 ± 14.51 | | 38.52 ± 12.30 | - | |  |
| Özbey Yücel et al., 2021 | ORTO-15 | Mean (SD) | 36.50 ± 3.81 | | 37.30 ± 3.85 | | 38.20 ± 3.78 | - | |  |
| Reynolds et al., 2023 | ORTO-15 | Mean (SD) | 38.90 ± 8.1 | | - | | 41.50 ± 6.30 | - | |  |
|  | ORTO-15 (≥35) | Prevalence, n (%) | 22 (31.4) | | - | | 44 (11.8) | - | |  |
| Ruiz & Quiles, 2021 | TOS | Mean (SD) | 4.97 ± 4.60 | | - | | 3.51 ± 3.35 | - | |  |
|  | ORTO-11 | Mean (SD) | 25.86 ± 5.45 | | - | | 28.40 ± 5.51 | - | |  |
|  | ORTO-11 (≥25) | Prevalence, n (%) | 39 (45) | | - | | 124 (27.68) | - | |  |
| Şentürk et al., 2022 | TOS | Mean (SD) | 5.82 ± 4.00 | | 5.73 ± 4.15 | | 5.03 ± 4.20 | - | |  |
| Strahler et al., 2018 | DOS (≥30) | Prevalence, n (%) | 2 (7.4) | | 4 (14.8) | | 15 (55.6) | - | |  |
| Tarsitano et al., 2022 | DOS (≥35) | Prevalence, n (%) | 254 (23.7) | | - | | 888 (30.3) | - | |  |

Abbreviations: BMI, body mass index; BOT, Bratman Test for Orthorexia; CI, confidence interval; DOS, Duesseldorf Orthorexia Scale; EHQ, Eating Habits Questionnaire; OR, Odds Ratio; ORTO-15/11/R, Questionnaire for the Diagnosis of Orthorexia; ON, Orthorexia Nervosa; SD, standard deviation; TOS, Teruel Orthorexia Scale.

**Supplementary Table 3.** List of the studies fully assessed for eligibility and excluded.

| **Wrong exposure (n=9)** |
| --- |
| - Maïano C, Aimé A, Almenara CA, et al. Psychometric properties of the Teruel Orthorexia Scale (TOS) among a French-Canadian adult sample. Eat Weight Disord 2022; 27: 3457–3467. - Davies G, Kalika E, Hussain M, et al. Exploring the association of body mass index, mindful and emotional eating, and orthorexia. Health Psychol Rep 2022; 11: 342–353. - Boutin C, Maïano C, Aimé A. Relation between orthorexia nervosa and healthy orthorexia: A latent profile analysis. Appetite; 194. Epub ahead of print 1 March 2024. DOI: 10.1016/J.APPET.2023.107165. - Bellikci Koyu E, Karaağaç Y, Öner BN. The association between food neophobia, bi-dimensional aspects of orthorexia, and anxiety among vegetarians and omnivores. Appetite; 197. Epub ahead of print 1 June 2024. DOI: 10.1016/J.APPET.2024.107303. - Coimbra M, Ferreira C. Making the leap from healthy to disordered eating: the role of intuitive and inflexible eating attitudes in orthorexic behaviours among women. Eat Weight Disord. 2021 Aug;26(6):1793-1800. - Plichta M, Jezewska-Zychowicz M. Orthorexic Tendency and Eating Disorders Symptoms in Polish Students: Examining Differences in Eating Behaviors. Nutrients; 12. Epub ahead of print 1 January 2020. DOI: 10.3390/NU12010218. - Roustaee R, Hajifaraji M, Djazayery A, et al. Major Dietary patterns among female adolescents with eating disorders: A factor analysis approach. Progress in Nutrition 2018; 20: 378–386. - Steinmann A, Ruf A, Ahrens KF, et al. Bacon, Brownie, or Broccoli? Beliefs about Stress-Relieving Foods and Their Relationship to Orthorexia Nervosa. Nutrients; 14. Epub ahead of print 1 September 2022. DOI: 10.3390/NU14183673. - Brytek-Matera A. The Polish version of the Düsseldorf Orthorexia Scale (PL-DOS) and its comparison with the English version of the DOS (E-DOS). Eating and Weight Disorders 2021; 26: 1223–1232. |
| **Wrong comparator (n=2)** |
| - Herranz Valera J, Acuña Ruiz P, Romero Valdespino B, et al. Prevalence of orthorexia nervosa among ashtanga yoga practitioners: a pilot study. Eat Weight Disord 2014; 19: 469–472. - Erkin Ö, Göl I. Determination of health status perception and orthorexia nervosa tendencies of Turkish yoga practitioners: A cross-sectional descriptive study. Progress in Nutrition 2019; 21: 105–112. |
| **Wrong outcome (n=44)** |
| - AL-Mohaithef M. Prevalence of vegan/vegetarian diet and eating behavior among Saudi adults and its correlation with body mass index: A cross-sectional study. Front Nutr; 9. Epub ahead of print September 15, 2022. DOI: 10.3389/FNUT.2022.966629. - Baş M, Karabudak E, Kiziltan G. Vegetarianism and eating disorders: association between eating attitudes and other psychological factors among Turkish adolescents. Appetite 2005; 44: 309-315. - Bardone-Cone AM, Fitzsimmons-Craft EE, Harney MB, et al. The inter-relationships between vegetarianism and eating disorders among females. J Acad Nutr Diet 2012; 112: 1247-1252. - Barrack MT, West J, Christopher M, et al. Disordered Eating Among a Diverse Sample of First-Year College Students. J Am Coll Nutr 2019; 38: 141-148. - Brytek-Matera A. Interaction between Vegetarian Versus Omnivorous Diet and Unhealthy Eating Patterns (Orthorexia Nervosa, Cognitive Restraint) and Body Mass Index in Adults. Nutrients 2020, Vol 12, Page 646 2020; 12: 646. - Brytek-Matera A. Restrained Eating and Vegan, Vegetarian and Omnivore Dietary Intakes. Nutrients 2020; 12: 1–16. - Brytek-Matera A, Czepczor-Bernat K, Jurzak H, et al. Strict health-oriented eating patterns (orthorexic eating behaviours) and their connection with a vegetarian and vegan diet. Eat Weight Disord 2019; 24: 441–452. - Collins M, Quinton S. The inter-relationship between diet, selflessness, and disordered eating in Australian women. Brain Behav; 10. Epub ahead of print September 1, 2020. DOI: 10.1002/BRB3.1774. - de Carvalho RC, Moreira JM. Níveis de Concordância com Regras Descritivas sobre Comportamentos Alimentares entre Veg(etari)anos. Psico-USF 2020; 25: 533-545. - Dorard G, Mathieu S. Vegetarian and omnivorous diets: A cross-sectional study of motivation, eating disorders, and body shape perception. Appetite; 156. Epub ahead of print January 1, 2021. DOI: 10.1016/J.APPET.2020.104972. - Estima CCP, Philippi ST, Leal GVS, et al. Vegetarianism and eating disorder risk behavior in adolescents from São Paulo, Brazil. Revista Espanola de Nutricion Humana y Dietetica 2012; 16: 94-99. - Fan J, Nagata JM, Cuccolo K, et al. Associations between dieting practices and eating disorder attitudes and behaviors: Results from the Canadian study of adolescent health behaviors. Eat Behav 2024; 54: 101886. - Fatima W, Ahmad LM. Prevalence of disordered eating attitudes among adolescent girls in Arar City, Kingdom of Saudi Arabia. Health Psychol Res; 6. Epub ahead of print November 29, 2018. DOI: 10.4081/HPR.2018.7444. - Fisak B, Peterson RD, Tantleff-Dunn S, et al. Challenging previous conceptions of vegetarianism and eating disorders. Eat Weight Disord 2006; 11: 195-200. - Forestell CA, Spaeth AM, Kane SA. To eat or not to eat red meat. A closer look at the relationship between restrained eating and vegetarianism in college females. Appetite 2012; 58: 319-325. - Heiss S, Boswell JF, Hormes JM. Confirmatory factor analysis of the Eating Disorder Examination-Questionnaire: A comparison of five factor solutions across vegan and omnivore participants. Int J Eat Disord 2018; 51: 418-428. - Heiss S, Coffino JA, Hormes JM. Eating and health behaviors in vegans compared to omnivores: Dispelling common myths. Appetite 2017; 118: 129-135. - Heiss S, Walker DC, Anderson DA, et al. Vegetarians and omnivores with diagnosed eating disorders exhibit no difference in symptomology: a retrospective clinical chart review. Eat Weight Disord 2021; 26: 1007-1012. - JANELLE KC, BARR SI. Nutrient intakes and eating behavior scores of vegetarian and nonvegetarian women. J Am Diet Assoc 1995; 95: 180-189. - Klopp SA, Heiss CJ, Smith HS. Self-reported vegetarianism may be a marker for college women at risk for disordered eating. J Am Diet Assoc 2003; 103: 745-747. - Köse G, Tayfur M, Karayigit R. BMI, Physical Activity, Sleep Quality, Eating Attitudes, Emotions: Which One is Affected by Mindful Eating? Progress in Nutrition 2021; 23: e2021002. - Lindeman M, Stark K, Latvala K. Vegetarianism and eating-disordered thinking. Eat Disord 2000; 8: 157-165. - Lindeman M. The state of mind of vegetarians: Psychological well-being or distress? Ecol Food Nutr 2002; 41: 75-86. - McLean CP, Kulkarni J, Sharp G. The 26-Item Eating Attitudes Test (EAT-26): Psychometric Properties and Factor Structure in Vegetarians and Vegans. Nutrients; 15. Epub ahead of print January 1, 2023. DOI: 10.3390/NU15020297. - McLean CP, Lavale A, Kulkarni J, et al. Psychometric properties and factor structure of the Eating Disorder Examination-Questionnaire in omnivores, vegetarians, and vegans. Body Image 2022; 43: 374-384. - McLean CP, Moeck EK, Sharp G, et al. Characteristics and clinical implications of the relationship between veganism and pathological eating behaviours. Eat Weight Disord 2022; 27: 1881-1886. - Michalak J, Zhang XC, Jacobi F. Vegetarian diet and mental disorders: results from a representative community survey. International Journal of Behavioral Nutrition and Physical Activity 2012; 9: 67. - Neumark-Sztainer D, Story M, Resnick MD, et al. Adolescent vegetarians. A behavioral profile of a school-based population in Minnesota. Arch Pediatr Adolesc Med 1997; 151: 833-838. - Norwood R, Cruwys T, Chachay VS, et al. The psychological characteristics of people consuming vegetarian, vegan, paleo, gluten free and weight loss dietary patterns. Obes Sci Pract 2019; 5: 148-158. - Parra-Fernández ML, Manzaneque-Cañadillas M, Onieva-Zafra MD, et al. Pathological Preoccupation with Healthy Eating (Orthorexia Nervosa) in a Spanish Sample with Vegetarian, Vegan, and Non-Vegetarian Dietary Patterns. Nutrients 2020; 12: 1–12. - Paslakis G, Richardson C, Nöhre M, et al. Author Correction: Prevalence and psychopathology of vegetarians and vegans - Results from a representative survey in Germany (Scientific Reports, (2020), 10, 1, (6840), 10.1038/s41598-020-63910-y). Sci Rep 2020; 10: 19811. - Perry CL, Mcguire MT, Neumark-Sztainer D, et al. Characteristics of vegetarian adolescents in a multiethnic urban population. Journal of Adolescent Health 2001; 29: 406-416. - Reuber H, Muschalla B. Dietary identity and embitterment among vegans, vegetarians and omnivores. Health Psychol Behav Med 2022; 10: 1038-1055. - Robinson-O'Brien R, Perry CL, Wall MM, et al. Adolescent and young adult vegetarianism: better dietary intake and weight outcomes but increased risk of disordered eating behaviors. J Am Diet Assoc 2009; 109: 648-655. - S M Gilbody SFK. Vegetarianism in young women: another means of weight control? - PubMed. Int J Eat Disord 1999; 87-90. - Sandri E, Sguanci M, Cantín Larumbe E, et al. Plant-Based Diets versus the Mediterranean Dietary Pattern and Their Socio-Demographic Determinants in the Spanish Population: Influence on Health and Lifestyle Habits. Nutrients; 16. Epub ahead of print April 25, 2024. DOI: 10.3390/NU16091278. - Sieke EH, Carlson JL, Lock J, et al. To meat or not to meat: disordered eating and vegetarian status in university students. Eat Weight Disord 2022; 27: 831-837. - Timko CA, Hormes JM, Chubski J. Will the real vegetarian please stand up? An investigation of dietary restraint and eating disorder symptoms in vegetarians versus non-vegetarians. Appetite 2012; 58: 982-990. - Turner-McGrievy GM, Wilcox S, Frongillo EA, et al. Differences in dietary acceptability, restraint, disinhibition, and hunger among African American participants randomized to either a vegan or omnivorous soul food diet. Appetite 2024; 196: 1-8. - Trautmann, J; Rau, S I.; Wilson, M A.; Walters, C. Vegetarian students in their first year of college: Are they at risk for restrictive or disordered eating behaviors?. College Student Journal 2008; 42: 2; 340-347. - Wiśniewska K, Okręglicka KM, Jaworski M, Nitsch-Osuch A. Plant-Based vs. Animal-Based Diets: Appetitive Traits and Dietary Patterns in Adults Based on Cross-Sectional Surveys. Nutrients. 2025 Feb 4;17(3):573. doi: 10.3390/nu17030573. PMID: 39940431; PMCID: PMC11819905. - Worsley A, Skrzypiec G. Teenage vegetarianism: Beauty or the beast? Nutrition Research 1997; 17: 391-404. - Zickgraf HF, Hazzard VM, O'Connor SM, et al. Examining vegetarianism, weight motivations, and eating disorder psychopathology among college students. Int J Eat Disord 2020; 53: 1506-1514. - Zuromski KL, Witte TK, Smith AR, et al. Increased prevalence of vegetarianism among women with eating pathology. Eat Behav 2015; 19: 24-27. |

**Supplementary Table 4.** Methodological quality of included studies^a^.

| **Reference** | **1** | **2** | **3** | **4** | **5** | **6** | **7** | **8** | **9** | **10** | **11** | **12** | **13** | **14** | **Score** | **Quality** |
| --- | --- | --- | --- | --- | --- | --- | --- | --- | --- | --- | --- | --- | --- | --- | --- | --- |
| Albery et al. (2025) | Y | Y | CD | Y | Y | N | N | NA | Y | N | Y | N | NA | N | 6 | Fair |
| Barnett et al. (2016) | Y | Y | CD | Y | N | N | N | NA | Y | N | Y | N | NA | N | 5 | Poor |
| Barthels et al. (2018) | Y | Y | CD | N | N | N | N | NA | N | N | Y | N | NA | N | 3 | Poor |
| Białek-Dratwa et al. (2024) | Y | Y | CD | Y | N | N | N | NA | N | N | Y | N | NA | N | 4 | Poor |
| Chard et al. (2019) | Y | Y | CD | Y | N | N | N | NA | N | N | Y | N | NA | N | 4 | Poor |
| Çiçekoğlu & Tunçay (2018) | Y | Y | CD | N | N | N | N | NA | N | N | Y | N | NA | N | 3 | Poor |
| Coimbra & Ferreira (2021) | Y | Y | Y | Y | N | N | N | NA | N | N | Y | N | NA | N | 5 | Poor |
| Dai & Leung (2024) | Y | Y | CD | N | Y | N | N | NA | Y | N | Y | N | NA | Y | 5 | Poor |
| Dell’Osso et al. (2016) | Y | Y | CD | Y | N | N | N | NA | N | N | Y | N | NA | N | 4 | Poor |
| Dell`Osso et al. (2022) | Y | Y | CD | Y | N | N | N | NA | N | N | Y | N | NA | N | 4 | Poor |
| Dittfeld et al. (2017) | Y | Y | CD | NR | N | N | N | NA | N | N | Y | N | NA | N | 3 | Poor |
| Dunn et al. (2017) | Y | Y | CD | Y | N | N | N | NA | N | N | Y | N | NA | Y | 5 | Poor |
| Ferrerira & Coimbra (2020) | Y | Y | CD | Y | N | N | N | NA | N | N | Y | N | NA | N | 4 | Poor |
| Gwiozdzik et al. (2022) | Y | Y | CD | Y | N | N | N | NA | N | N | Y | N | NA | N | 4 | Poor |
| Hessler et al. (2021) | Y | Y | Y | Y | N | N | N | NA | Y | N | Y | N | NA | Y | 7 | Fair |
| Heiss et al. (2019) | Y | Y | CD | Y | N | N | N | NA | Y | N | Y | N | NA | Y | 6 | Fair |
| Luck‑Sikorski et al. (2019) | Y | Y | N | Y | N | N | N | NA | N | N | Y | N | NA | Y | 5 | Poor |
| Missbach et al. (2015) | Y | Y | Y | N | N | N | N | NA | N | N | Y | N | NA | Y | 5 | Poor |
| Novara et al. (2022) | Y | Y | N | Y | N | N | N | NA | Y | Y | Y | N | NA | Y | 7 | Fair |
| Oberle, De Nadai & Madrid (2021) | Y | Y | CD | N | N | N | N | NA | Y | N | Y | N | NA | Y | 5 | Poor |
| Özbey Yücel et al. (2021) | Y | Y | CD | N | N | N | N | NA | N | N | Y | N | NA | Y | 4 | Poor |
| Reynolds et al. (2023) | Y | Y | CD | Y | Y | N | N | NA | Y | N | Y | N | NA | N | 5 | Poor |
| Ruiz & Quiles (2021) | Y | Y | CD | Y | N | N | N | NA | N | N | Y | N | NA | N | 4 | Poor |
| Şentürk et al. (2022) | Y | Y | CD | Y | N | N | N | NA | N | N | Y | N | NA | N | 4 | Poor |
| Strahler et al. (2018) | Y | Y | CD | N | N | N | N | NA | Y | N | Y | N | NA | N | 4 | Poor |
| Tarsitano et al. (2022) | Y | Y | CD | N | N | N | N | NA | N | N | Y | N | NA | Y | 4 | Poor |

^a^ Numbers represent the questions included in the National Institutes of Health’s Quality Assessment Tool for Observational Cohort and Cross-Sectional Studies: 1. Question clear? 2. Population clearly defined? 3. >50% participants? 4. Recruitment populations consistent? 5. Sample size justified? 6. Exposure assessed prior to outcome? 7. Sufficient timeframe? 8. Different exposure levels? 9. Valid exposure? 10. Repeated exposure assessment? 11. Valid outcomes? 12. Outcome assessors blinded? 13. Loss to follow up <20%? 14. Confounders adjusted for? Item 8 was considered not applicable for dichotomous exposure. Item 13 was considered not applicable for cross sectional studies. Each cross-sectional study was rated as good, fair, or poor according to the National Institutes of Health’s Quality Assessment Tool for Observational Cohort and Cross-Sectional quality rating guide. Abbreviations: CD: cannot determine; N, no; NA, not applicable; NR, not reported; Y, yes.

**Supplementary Table 5.** Subgroup analyses for the cross-sectional associations between vegetarian and/or vegan vs. omnivorous diets and orthorexia nervosa symptoms.

| **MA of SMDs** | **n** | **SMD (95% CI)** | **p** | **I^2^ (%)** | **p _subgroup_** |
| --- | --- | --- | --- | --- | --- |
| **Diet^a^** |  |  |  |  | 0.855 |
| Vegetarian (vs. omnivorous) | 12 | 0.455 (0.278; 0.663) | < 0.001 | 79.8 |  |
| Vegan (vs. omnivorous) | 11 | 0.421 (0.095; 0.746) | 0.003 | 87.8 |  |
| **Scale** |  |  |  |  | < 0.001 |
| ORTO-15/11/R | 11 | 0.382 (0.257; 0.507) | < 0.001 | 38.4 |  |
| DOS | 6 | 0.765 (0.648; 0.881) | < 0.001 | 7.5 |  |
| **Region^b^** |  |  |  |  | 0.838 |
| Europe | 11 | 0.509 (0.347; 0.672) | < 0.001 | 70.7 |  |
| North America (USA) | 5 | 0.547 (0.223; 0.871) | < 0.001 | 83.8 |  |

^a^ Studies that did not report data on vegetarians and vegans separately were excluded from the analysis.

^b^ Some regions could not be included because there were not enough studies that included them.

Abbreviations: CI, confidence interval; DOS, Duesseldorf Orthorexia Scale; MA, meta-analysis; ORTO-15/11/R, Questionnaire for the Diagnosis of Orthorexia; SMD, standardized mean difference.

**Supplementary Table 6.** Meta-regression analyses for the cross-sectional associations between vegetarian and/or vegan vs. omnivorous diets and orthorexia nervosa symptoms.^a^

| **Covariates** |  | **Coefficients** | | | **Heterogeneity** | | | **Test of residual heterogeneity** | | |
| --- | --- | --- | --- | --- | --- | --- | --- | --- | --- | --- |
| **MA of SMDs** | **n** | **Estimate** | **SE** | ***p*** | ***I*^2^ (%)** | **H^2^** | **R^2^ (%)** | **Q** | **df** | ***p*** |
| Age (mean years) | 17 | -0.001 | 0.013 | 0.912 | 87.8 | 8.2 | 0.0 | 80.4 | 15 | <0.001 |
| Sex (% female) | 20 | 0.004 | 0.004 | 0.428 | 87.0 | 7.7 | 0.0 | 99.7 | 18 | <0.001 |
| BMI (mean kg/m^2^) | 9 | 0.002 | 0.053 | 0.977 | 78.1 | 4.6 | 0.0 | 42.1 | 7 | <0.001 |
| Educational Level | 13 | -0.004 | 0.003 | 0.171 | 80.8 | 5.2 | 8.6 | 35.1 | 11 | <0.002 |
| **MA of ORs** |  |  |  |  |  |  |  |  |  |  |
| Age (mean years) | 10 | 0.027 | 0.033 | 0.409 | 93.8 | 16.1 | 0.0 | 135.7 | 8 | <0.001 |
| Sex (% female) | 11 | -0.018 | 0.011 | 0.106 | 87.2 | 7.8 | 16.9 | 57.4 | 9 | <0.001 |

^a^ Univariate random-effects meta-regression models were used to estimate the proportion of between-study heterogeneity explained by each covariate (for pooled standardized mean differences: mean age –ranging from 20.9 to 43.4 years–, percentage of female –ranging from 40.6 to 100–, and body mass index –ranging from 22.6 to 27.1 kg/m^2^–, and educational level (percentage of university degree) –ranging from 36.6 to 100–; for pooled odds ratios: mean age –ranging from 19.6 to 50.6 years– and percentage of female –ranging from 40.6 to 100–), as well as the change in the effect size estimates for each 1-unit change in the characteristic included as predictors in the model.

n represents the number of studies included in each meta-regression model.

Abbreviations: BMI, body mass index; MA, meta-analysis; OR, odds ratio; SE, standard error; SMD, standardized mean difference.

**Supplementary Table 7.** Sensitivity analyses excluding studies one by one.

| **Reference removed** | **SMD** | **LL** | **UL** | **I^2^** |
| --- | --- | --- | --- | --- |
| All studies | 0.462 | 0.325 | 0.598 | 81.0 % |
| Albery et al. (2025) | 0.493 | 0.368 | 0.619 | 79.2 % |
| Barnett et al. (2016) | 0.458 | 0.315 | 0.601 | 82.0% |
| Barthels et al. (2018) | 0.439 | 0.303 | 0.575 | 79.1% |
| Białek-Dratwa et al. (2024) | 0.463 | 0.320 | 0.607 | 82.0% |
| Çiçekoğlu & Tunçay, (2018) | 0.485 | 0.357 | 0.613 | 80.8% |
| Coimbra & Ferreira, (2021) | 0.449 | 0.307 | 0.590 | 81.2% |
| Dai & Leung, (2024) | 0.435 | 0.303 | 0.567 | 78.3% |
| Dell`Osso et al. (2022) | 0.463 | 0.319 | 0.606 | 82.0% |
| Dell’ Osso et al. (2016) | 0.457 | 0.313 | 0.601 | 81.7% |
| Dunn et al. (2016) | 0.485 | 0.351 | 0.618 | 80.6% |
| Ferrerira & Coimbra, (2020) | 0.450 | 0.308 | 0.592 | 81.3% |
| Heiss et al. (2019) | 0.462 | 0.319 | 0.606 | 82.0% |
| Hessler et al. (2020) | 0.452 | 0.310 | 0.594 | 81.7% |
| Missbach et al. (2015) | 0.464 | 0.320 | 0.608 | 82.0% |
| Novara et al. (2022) | 0.456 | 0.319 | 0.594 | 81.9% |
| Oberle, De Nadai & Madrid, (2020) | 0.444 | 0.304 | 0.584 | 80.2% |
| Özbey Yücel et al. (2021) | 0.467 | 0.324 | 0.611 | 81.6% |
| Reynolds et al. (2023) | 0.465 | 0.321 | 0.608 | 82.0% |
| Ruiz & Quiles, (2021) | 0.463 | 0.319 | 0.607 | 82.0% |
| Şentürk et al. (2022) | 0.480 | 0.339 | 0.620 | 76.2% |
| **Reference removed** | OR | ***LL*** | ***UL*** | ***I^2^*** |
| All studies | 1.986 | 1.213 | 3.252 | 92.8% |
| Białek-Dratwa et al. (2024) | 1.946 | 1.126 | 3.366 | 93.4% |
| Chard et al. (2018) | 1.876 | 1.114 | 3.160 | 93.3% |
| Dell’ Osso et al. (2016) | 1.913 | 1.106 | 3.309 | 91.1% |
| Dittfeld et al. (2017) | 2.179 | 1.519 | 3.128 | 93.3% |
| Gwiozdzik et al. (2022) | 2.027 | 1.171 | 3.508 | 93.5% |
| Heiss et al. (2019) | 1.840 | 1.090 | 3.106 | 92.6% |
| Luck-Sikorski et al. (2018) | 1.864 | 1.100 | 3.159 | 93.1% |
| Reynolds et al. (2023) | 1.870 | 1.098 | 3.185 | 93.0% |
| Ruiz & Quiles, (2021) | 1.959 | 1.130 | 3.396 | 93.3% |
| Strahler et al. (2018) | 2.051 | 1.198 | 3.512 | 93.5% |
| Tarsitano et al. (2022) | 2.270 | 1.381 | 3.733 | 55.0% |

Abbreviations: LL, low limit; OR, odds ratio; SMD, standardized mean differences; UL, upper limit.

**Supplementary Table 8.** Meta-bias for the cross-sectional associations between vegetarian and/or vegan vs. omnivorous diets and orthorexia nervosa symptoms.

| **Meta-analysis (ES)** | **n** | **Estimate (SE)** | **p** |
| --- | --- | --- | --- |
| SMD | 20 | 0.407 (1.322) | 0.762 |
| OR | 11 | 3.004 (1.753) | 0.121 |

*n* represents the number of studies included in each analysis.

Abbreviations: ES, effect size; OR: odds ratio; SE, standard error; SMD, standardized mean difference

**Supplementary Figure 1.** Meta-regression models by age (A), sex (B), body mass index (C), and educational level (D) for the cross-sectional associations between vegetarian and/or vegan vs. omnivorous diets and orthorexia nervosa symptoms.

**A**
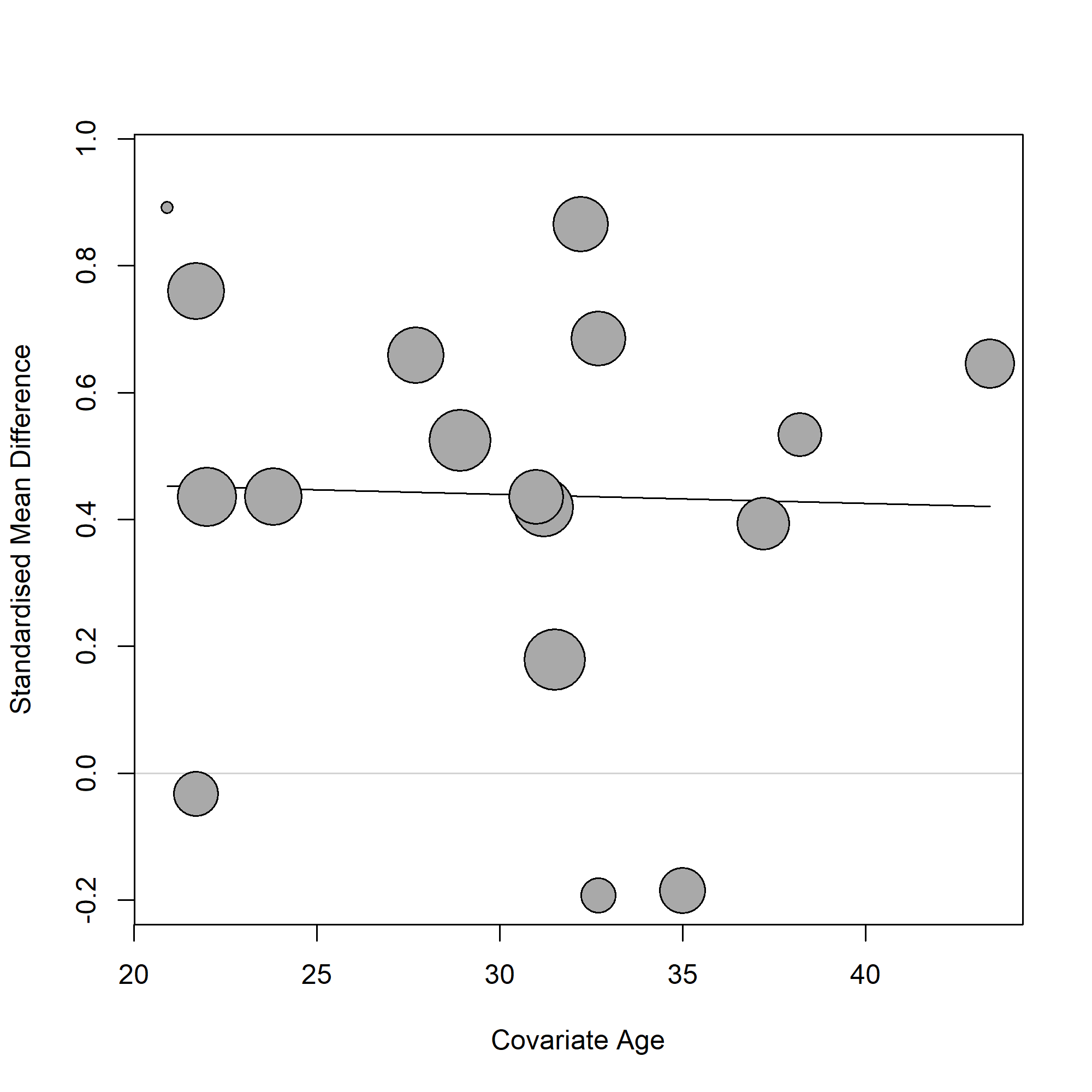


**B**
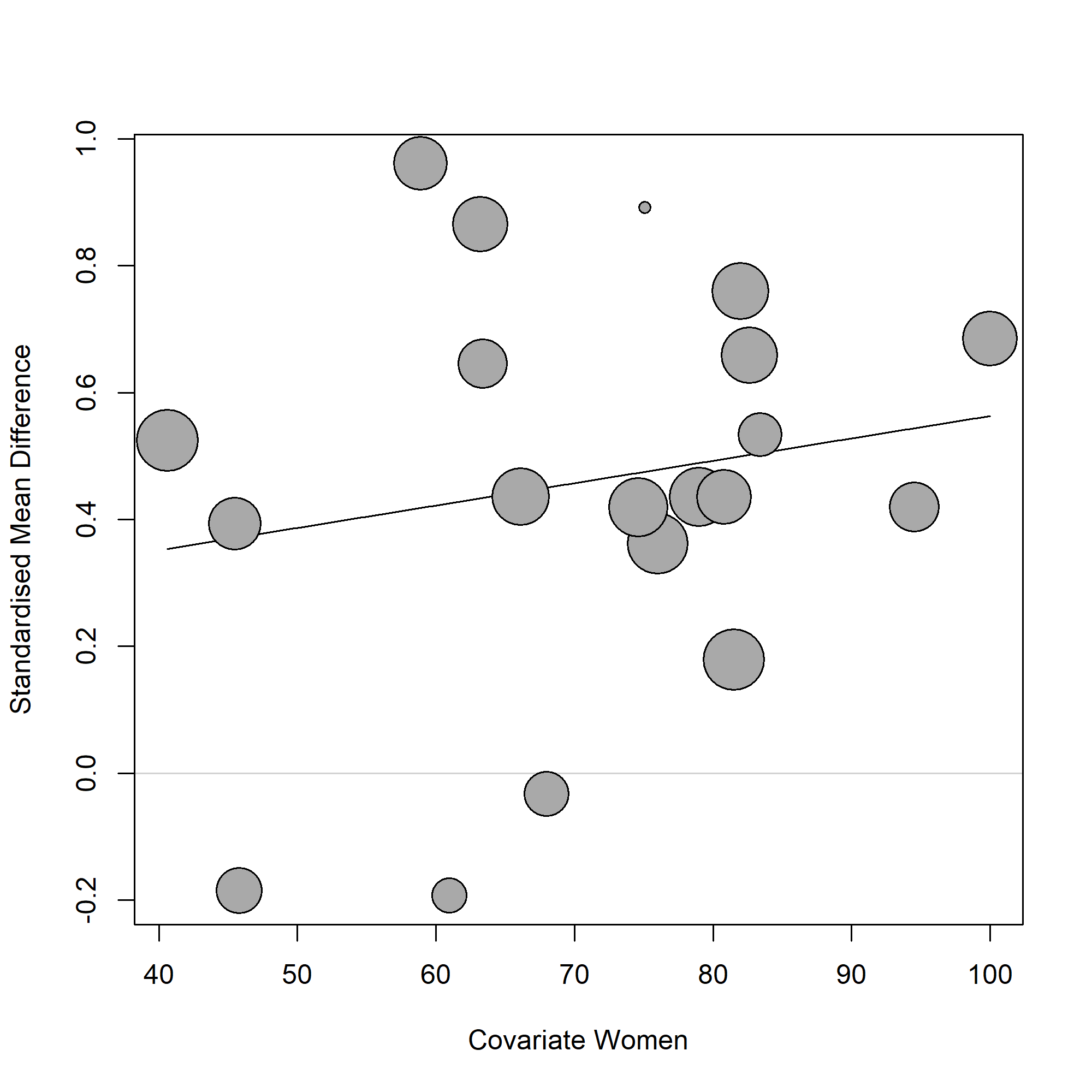


**C**
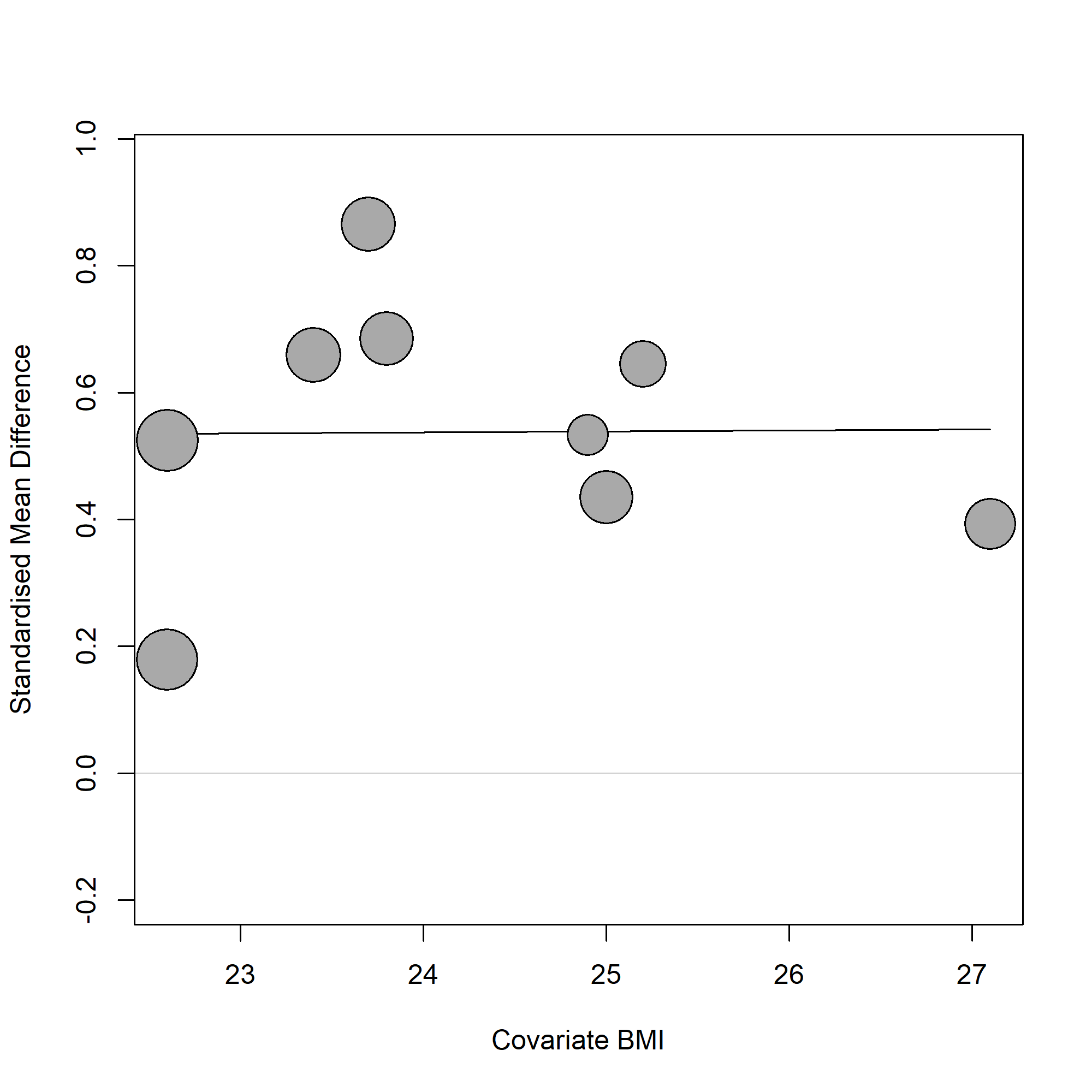


**D**
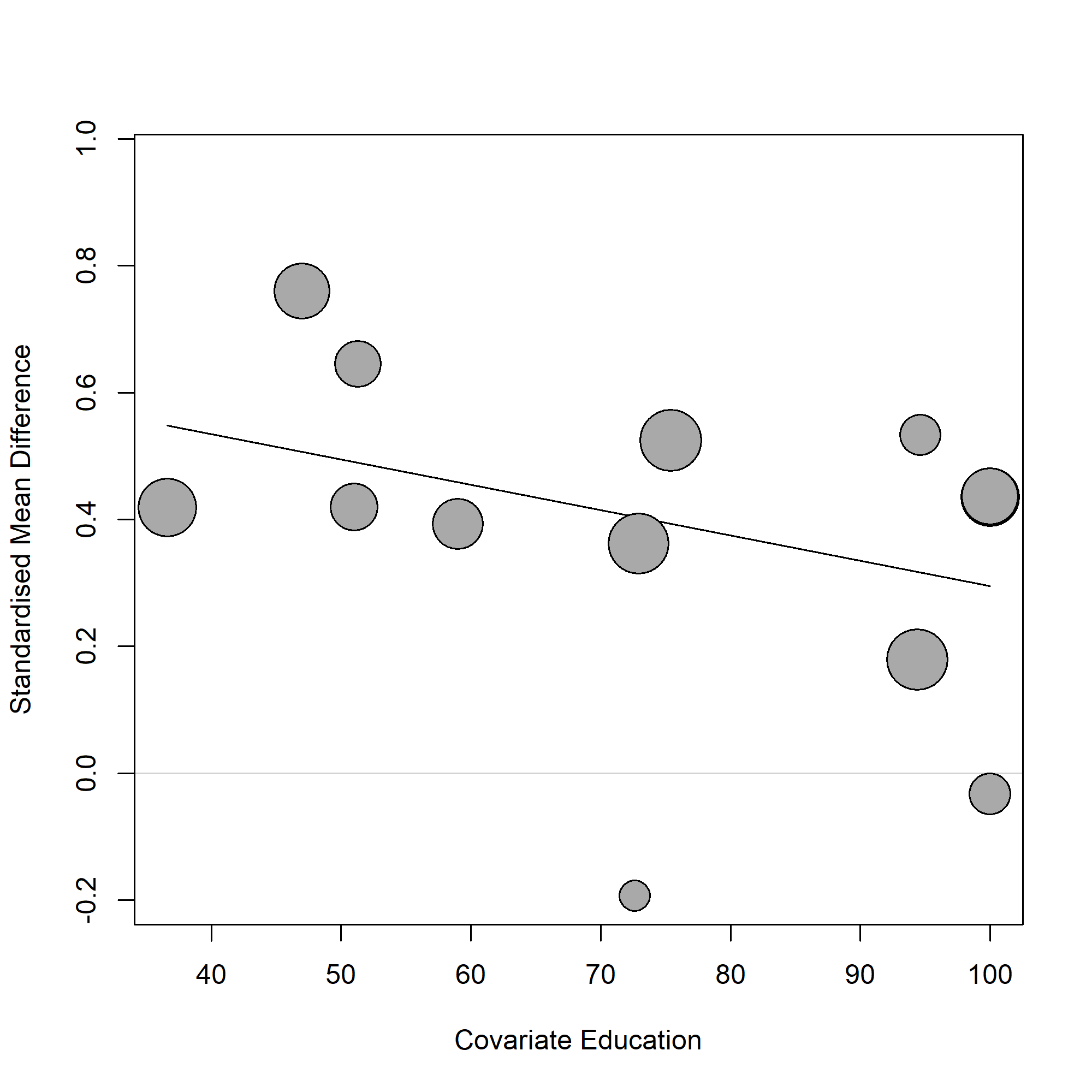


**Supplementary Figure 2.** Publication bias from cross-sectional meta-analyses of standardized mean differences (A) and odds ratio (B).

**B**
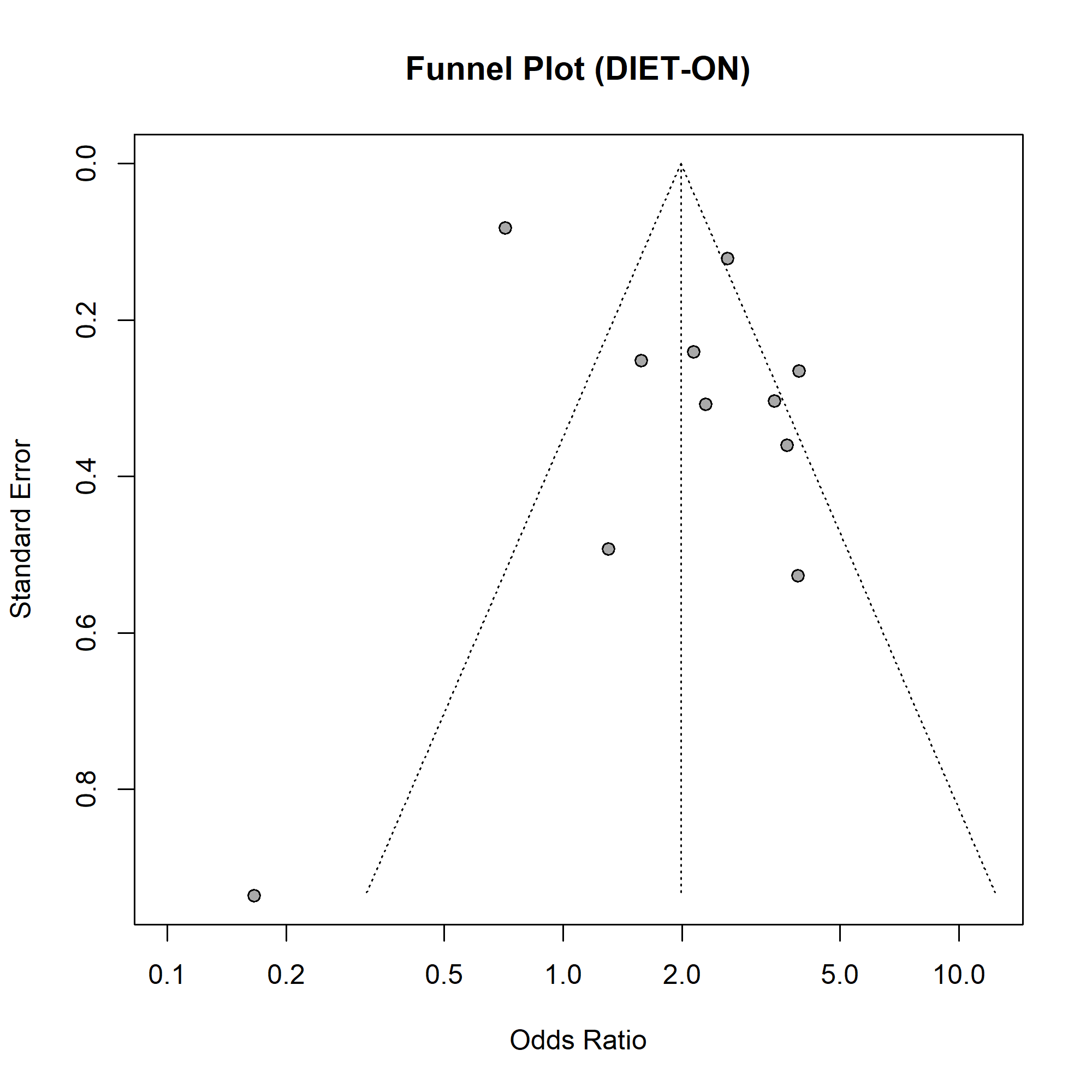


**A**
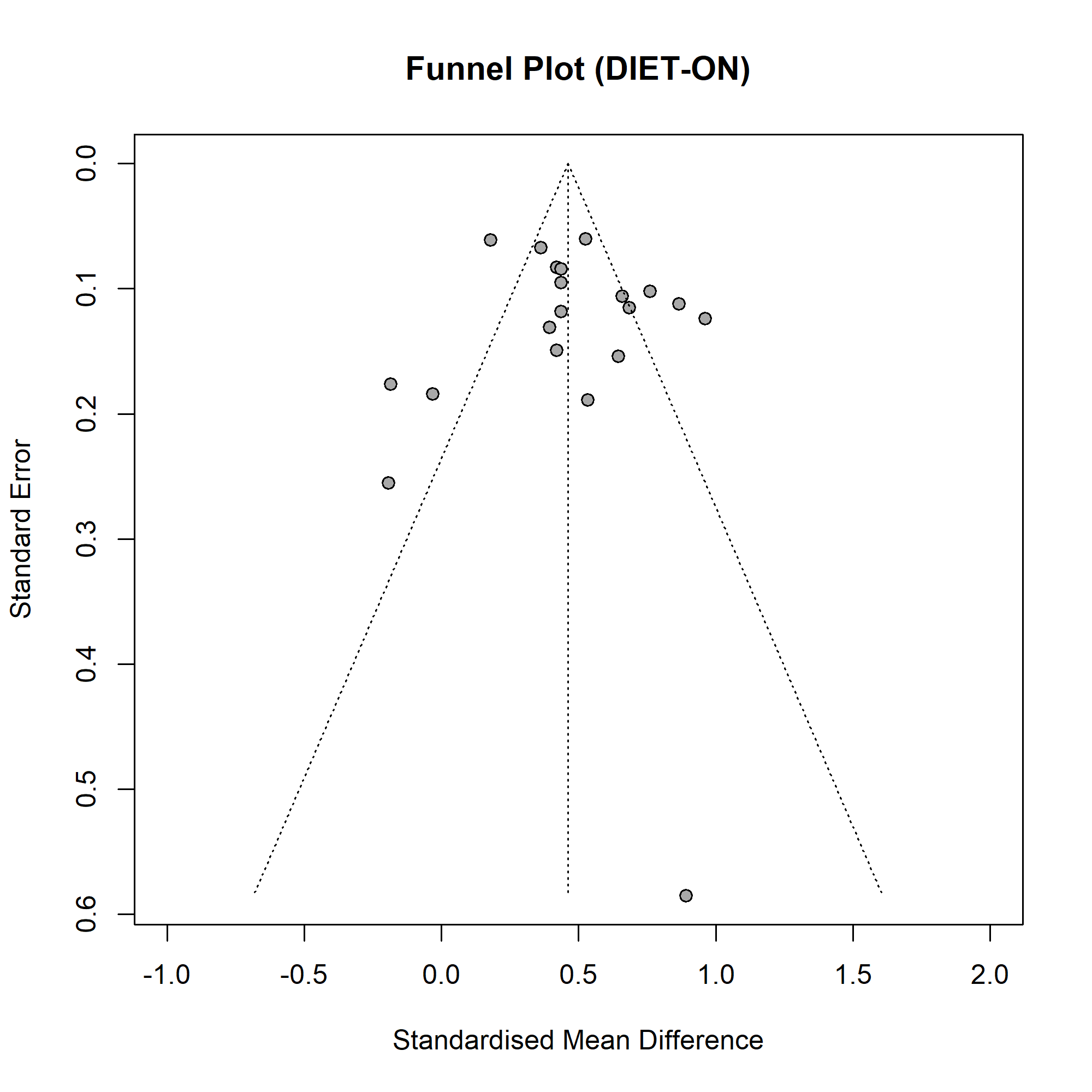

Supplement: Supplementary file 1 — Table S1: Detailed information on search strategies. Table S2: Effect sizes, main results, and covariate adjustments of included studies. Table S3: List of the studies fully assessed for eligibility and excluded. Table S4:. Methodological quality of included studiesa. Table S5: Subgroup analyses for the cross‐sectional associations between vegetarian and/or vegan versus omnivorous diets and orthorexia nervosa symptoms. Table S6: Meta‐regression analyses for the cross‐sectional associations between vegetarian and/or vegan versus omnivorous diets and orthorexia nervosa symptoms. a Table S7:. Sensitivity analyses excluding studies one by one. Table S8: Meta‐bias for the cross‐sectional associations between vegetarian and/or vegan versus omnivorous diets and orthorexia nervosa symptoms. Figure S1: Meta‐regression models by age (A), sex (B), body mass index (C), and educational level (D) for the cross‐sectional associations between vegetarian and/or vegan versus omnivorous diets and orthorexia nervosa symptoms. Figure S2: Publication bias from cross‐sectional meta‐analyses of standardized mean differences (A) and odds ratio (B). [file EAT-59-409-s001.docx]
